# Targeted Therapy in Breast Cancer: Advantages and Advancements of Antibody–Drug Conjugates, a Type of Chemo-Biologic Hybrid Drugs

**DOI:** 10.3390/cancers16203517

**Published:** 2024-10-17

**Authors:** Attrayo Mukherjee, Debasish Bandyopadhyay

**Affiliations:** 1 School of Biotechnology, Kalinga Institute of Industrial Technology (KIIT), Patia, Bhubaneswar 751024, Odisha, India; attrayomukh@gmail.com; 2 School of Integrative Biological and Chemical Sciences (SIBCS), University of Texas Rio Grande Valley, Edinburg, TX 78539, USA; 3 School of Earth, Environmental, and Marine Sciences (SEEMS), University of Texas Rio Grande Valley, Edinburg, TX 78539, USA

**Keywords:** breast cancer, antibody–drug conjugate, chemo-biologic drugs, target specificity, HER2+ breast cancer, triple-negative breast cancer, mechanism of action, drug-to-antibody ratio (DAR)

## Abstract

Cancer is a significant health challenge globally, with millions of people affected every year, resulting in high morbidity and mortality. Although other treatment options are available with limitations, chemotherapy, either standalone or combined with other therapeutic procedures, is the most commonly used practice of treating cancer. In chemotherapy, cancer cells/malignant tumors are targeted; however, due to less target specificity, along with malignant cells, normal cells are also affected, which leads to various off-target effects (side effects) that impact the patient quality of life. Out of all the different types of cancers, breast cancer is the most common type of cancer in humans worldwide. Current anticancer drug discovery research aims to develop therapeutics with higher potency and lower toxicity, which is only possible through target-specific therapy. Antibody–drug conjugates (ADCs) are explicitly designed to target malignant tumors and minimize off-target effects by reducing systemic cytotoxicity. Several ADCs have been approved for clinical use and have shown moderate to good efficacy so far. Considering various aspects, chemotherapy and ADCs are useful in treating cancer. However, ADCs provide a more focused and less toxic approach, which is especially helpful in cases where resistance to chemotherapy (drug resistance) occurs and in the type of malignancies in which specific antigens are overexpressed. Ongoing ADC research aims to develop more target-specific cancer treatments. In short, this study presents a concise overview of ADCs specific to breast cancer treatment. This study provides insight into the classifications, mechanisms of action, structural aspects, and clinical trial phases (current status) of these chemo-biologic drugs (ADCs).

## Simple Summary

Cancer is one of the leading causes of death, with one out of six deaths worldwide. Breast cancer has the highest occurrence of other cancers, although, in terms of the number of deaths, breast cancer ranks fifth among all cancers. Depending on the nature of the cells and the associated cellular environment, different types of breast cancer are possible. Many breast cancer types are highly challenging to treat, and even if treatable, the treatments involve several side effects. Antibody–drug conjugates (ADCs) comprise monoclonal antibodies that explicitly target the tumor-specific antigens and cytotoxic drugs (mainly small molecules) that act as payloads to kill the malignant cells/tumor. As a result, the healthy cells are not affected by the toxic drugs, which potentially reduces the side effects. These target-specific chemo-biologic drugs (ADCs) have shed new light on hopes in cancer therapy. Currently, three ADCs have been approved for breast cancer treatment, and many are in clinical trials. A detailed discussion on the biochemical nature, mechanism of action, and current status of ADCs in breast cancer treatments has been accomplished in this article. While we made every effort to include all the related developments in this field, any omission is truly unintentional.

## 1. Introduction

According to the World Health Organization (WHO), breast cancer is the most common type of cancer in humans, accounting for 2.26 million incidences and 685,000 deaths in 2020 [1]. Breast cancer is caused by aberrant breast cells that proliferate and develop into tumors. Tumors have the potential to spread within the human body and prove to be lethal if ignored. Breast milk ducts and the milk-producing lobules are the initial sites where the cancer cells start proliferation. Women without any particular risk factors apart from age and sex contribute to nearly fifty percent of all cases of breast cancer. The greatest risk factor for breast cancer is the female gender. Women acquire breast cancer in approximately 99% of cases, while men acquire breast cancer in 0.5–1% of cases. Breast cancer care in men is based on the same concepts as in women. Age, weight gain, heavy alcohol consumption, exposure to radiation history, a background of breast cancer in the family, reproductive history (which includes age during the initial pregnancy and menstruation onset), use of tobacco products, and postmenopausal hormonal therapy are some of the criteria that raise the likelihood of developing breast cancer [2]. 

Chemotherapy is the primary method to treat a variety of cancers, whereas immunotherapy, radiation, and surgery are the additional categories of conventional therapeutic techniques. Small cytotoxic medications have been used to accomplish the most impressive feats, but they also have a number of disadvantages that have hampered their effectiveness, such as a significant off-tumor effect and a poor therapeutic index. Primarily due to the non-specific impact of chemotherapeutic medications on rapidly dividing normal cells, patients typically had an elevated risk of severe adverse effects as a result of the low efficiency of chemotherapy. As a result, one of the hottest subjects in the industry is the development of novel chemical agents that preferentially destroy tumor-derived cells while possessing improved therapeutic efficiency [3]. 

The targeted therapy revolution began with the German scientist Paul Ehrlich’s discovery of the “Magic Bullet Theory.” The research and development of antibody–drug conjugates (ADCs), specifically in oncology, has provided a pharmaceutical solution to the most pressing issue, which is the requirement for anticancer treatment that targets tumor cells with high specificity and efficiency. The remarkable progress in monoclonal antibody research has made it possible to conjugate a variety of cytotoxic chemicals, including immunotoxins, cytotoxic medicines, and radiopharmaceuticals, to the antibody, which leads to a high level of targetability and selectivity to the particular antigen that is expressed on malignant cells [4]. The bioactivity of the new generation of ADCs not only includes the selective tumor delivery of highly cytotoxic payloads but also encompasses tumors that are thought to be naturally immune against the payload class of drugs due to the intricate interactions that each component has with the tumor and its microenvironment. As the linker pairing does not alter the antigen-binding site, the antibody backbone retains its properties for modulating tumor targets. Additionally, it functions as an immune effector, which triggers complement-dependent cytotoxicity (CDC), antibody-dependent cellular phagocytosis, and antibody-dependent cellular cytotoxicity (ADCC) through the Fc region. Currently, 14 ADCs for solid tumors and hematologic malignancies have been authorized in various countries; three of these are approved to treat early-stage or metastatic breast cancer (BC). However, ADCs have also given rise to novel toxicities, which can negatively impact patients’ standard of living and necessitate knowledge for the best possible care. This account focuses on the current status and future aspects of ADCs in breast cancer (BC) treatment [5].

## 2. Types of Breast Cancer

Depending on the cell types, histological profiles, invasiveness, presence (or absence) of estrogen and progesterone receptors (ER and PR), human epidermal growth factor receptor 2 (HER2), and some other factors, BC can be classified in many ways. A glimpse of some common types of BC is given in the following.

### 2.1. HER2+ Breast Cancer

The HER2 (also known as erythroblastic oncogene B2 or ErBb2) intrinsic tyrosine kinase receptor transduces biochemical signals across the cell membrane. In HER2-positive breast cancer, the *HER2* gene is highly overexpressed, resulting in uncontrolled cell division. HER2 belongs to the EGFR (epidermal growth factor receptor) family that comprises four members: (i) HER1 or ErBb1; (ii) HER2 or ErBb2; (iii) HER3 or ErBb3; (iv) HER4 or ErBb4 [6]. The *HER2* (or *ErBb2*) gene is amplified in this subgroup of BC. *ErBb2,* found on chromosome 17q21, was discovered in the 1980s when human breast cancer showed signs of overexpression. The human epidermal growth factor receptor 2-positive (HER2+) breast cancer accounts for 20–30% of all breast cancers [7].

### 2.2. Luminal Breast Cancer

Luminal breast cancers, which are ER-positive tumors, account for approximately 70% of all incidences of BC in Western countries. Luminal-like malignancies typically manifest as invasive breast cancer (IBC) without a specific subtype, although they can also occasionally develop into invasive micropapillary, invasive lobular, invasive cribriform, mucinous, and tubular carcinomas. Proliferation-related pathways and luminal-regulated pathways are the two primary biological mechanisms that differentiate luminal-like tumors into luminal A and luminal B variants, each with a distinct clinical manifestation [8].

#### 2.2.1. Luminal A

The presence of estrogen receptors (ER) and/or progesterone receptors (PR) and the lack of HER2 (HER2 negative) are characteristics of luminal A tumors. This subtype is characterized by the activation of genes by ER transcription factors that are specific to the luminal epithelium lining the mammary ducts. Additionally, it exhibits decreased gene expression, which is involved in cell division. They are easy to diagnose, inferior, and grow slowly [8].

#### 2.2.2. Luminal B

The prognosis for luminal B tumors is lower than luminal A due to its greater tumor grade. In addition to being ER positive, they could be PR-negative and HER2 positive. Furthermore, luminal B tumors exhibit elevated expression levels of genes linked to proliferation, such as *MKI67* and *AURKA*. The *PR* and *FOXA1* genes and proteins, which are distinctive of the luminal epithelium, are expressed less frequently among this subtype than the *ER*. Estrogen receptors differentiate between luminal and non-luminal diseases since they appear similarly in A and B subtypes [8].

### 2.3. Triple-Negative Breast Cancer

Triple-negative breast cancers (TNBC) or basal-like breast cancers are basically of two subtypes, BL1 and BL2, and account for approximately 15% of all BCs. In the context of gene expression patterns and IHC analysis, nearly all TNBCs are of the basal-like subtype, and many basal-like breast tumors are triple negative [9]. Gene expression profiling is the basis for identifying different types of basal-like breast cancer. Despite their seeming similarity, however, a 30% discrepancy lies between both groups. Basal-like breast cancers are distinguished by a high expression of *CK5*, *CK14*, *caveolin-1*, *caix*, *p63*, and *EGFR* (epidermal growth factor receptor gene)/*HER1*, which focus mainly on the basal/myoepithelial cell components of the mammary gland, along with reduced expression of *PR*, *ER*, and *HER2* [10]. Although TNBC accounts for approximately 15% of all BCs, *BRCA1* or *BRCA2* germline mutations (inherited mutations) are present in approximately 80% of BCs, and a child of a parent with either *BRCA1* or *BRCA2* mutation has a 50% possibility to inherit that mutation. TNBC is frequently linked to a poorer diagnosis and tends to be invasive and difficult to treat by chemotherapy [8,9].

### 2.4. Claudin-Low Breast Cancer

A unique subgroup known as claudin-low breast cancer (CLBC) was discovered in 2007 and is distinguished by intrinsic molecular characteristics and clinical traits. CLBC is characterized by the strong expression of mesenchymal markers (*Vimentin*, *Snai 1/2*, *Zeb 1/2*, *Twist 1/2*), stem cell-like characteristics (*ALDH1*), and low expression of claudin (particularly *claudin 3*, *4*, and *7*), *E-cadherin*, and *HER2*. This aggressive subtype usually appears after menopause, with a higher histological grade and greater invasiveness, and involves lymph nodes. Reduced claudin expression causes damaged tight junctions, which activate signaling pathways that encourage the growth of tumors and contribute to the pathophysiology of CLBC. Effective treatments are still investigational, lacking clinical trials or established animal research, even with recognized medicinal targets. Poor response to existing therapy is a common feature of CLBC, as evidenced by low rates of histological full remission and a dismal prognosis. To improve knowledge and therapy choices for this challenging subtype of breast cancer, more research is essential [11]. 

Apart from the above, based on the invasiveness [12], important types of BCs include (i) invasive ductal carcinoma (IDC), which is a type of adenocarcinoma that accounts for 70–80% of all BCs. IDC starts in the epithelial cells of the milk ducts and spreads aggressively. (ii) Invasive lobular carcinoma (ILC), a type of adenocarcinoma that starts in the epithelial cells of the breast lobules which are glands that are present in the breast and make milk). ILC accounts for approximately 12% of all BCs. (iii) Ductal carcinoma in situ (DCIS or intraductal carcinoma), which is an early stage (precancer, non-invasive) of BC that accounts for 20% (one in five) new BCs with a high recovery rate [13]. (iv) Inflammatory breast cancer in which malignant cells block the vessels present in the skin and cause the breast to look ‘inflamed.’ This type of BC accounts for 1 to 5% of all BCs and invades aggressively. Alternatively, some rare types of BCs include (i) Paget disease of the breast, which starts in the ducts and spreads to the skin of the nipple and areola (the dark circle), accounting for 1–3% of all BCs; (ii) phyllodes tumor, which develops in the breast connective tissues (stroma) and is rarely malignant (mostly benign); (iii) angiosarcoma, which grows in the cells that are lined with blood or lymph vessels, accounting for less than 1% of all BCs; (iv) metaplastic BC, consisting of various cell types, is aggressive and accounts for approximately 1% of all BCs; (v) colloid or mucinous carcinoma, which develops in the mucus-producing cells and accounts for 1–2% of all BCs; (vi) male breast cancer, which develops in the men, can be of invasive (IDC) or non-invasive (DCIS), and accounts for approximately 1% of all BCs. The major reasons may be the inheritance of BRCA1 and BRCA2 mutations and/or the presence of an extra X chromosome that increases estrogen levels and lowers androgen levels (Klinefelter syndrome), etc. [14].

## 3. Targeted Therapy

Depending on the cell types, histological profiles, invasiveness, presence (or absence) of estrogen and progesterone receptors (ER and PR), human epidermal growth factor receptor 2 (HER2), and some other factors, BC can be classified in many ways. Below is a glimpse of some common types of BC.

### 3.1. Importance of Targeted Therapy: Why Antibody–Drug Conjugates (ADCs)?

Although some targeted drug delivery methods are in the investigation stage, antibody–drug conjugates are the best-recognized method for precisely delivering drugs to malignant tumors. The significant benefits [15] of ADCs include that they can (i) deliver small-molecule therapeutics to inhibit specific proteins that help cancer cell proliferation, tumor angiogenesis, and metastasize malignant cells to other parts of the body; (ii) boost the immune system to perform better in destroying cancer cells and/or to mark the malignant cells, making it easier to be identified and killed by the immune system; (iii) inhibit proteins associated with the signals that cause uncontrolled cell division; (iv) act as angiogenesis inhibitors by preventing signals that help to form new blood vessels in the tumors; (v) accelerate apoptosis (a programmed cell death procedure) of the cancer cells; (vi) stop the growth of hormone-sensitive tumors (for example, ER+ tumors) by preventing the generation of required hormone in the body, or even form in the body, by deactivating them from their actions. This process is commonly known as hormone therapy. 

### 3.2. Molecular Structure and General Mechanism of Antibody–Drug Conjugates

**Figure 1:**
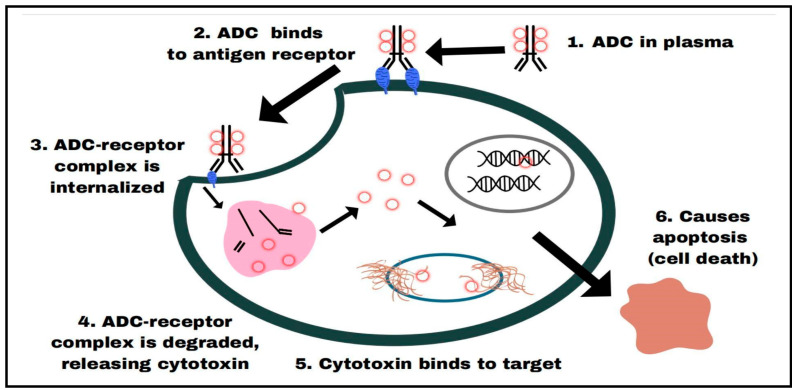
Representative mechanistic pathway of ADC in cancer cells.

An ADC is a product created when a monoclonal antibody and a small-molecule medicine (payload) are covalently coupled via a linker in most cases (tri-partite ADC). Alternatively, mAb (monoclonal antibodies, which are heterodimeric proteins with an approximate molecular weight of 150 kDa) can directly be attached to a small molecule (bi-partite ADC). The general physical and chemical characteristics (mechanisms of action), effectiveness, and potential drawbacks of ADC medications are all defined in this review. At present, clinically used, intact therapeutic antibodies are immunoglobulins (IgG), which are glycoproteins with a Y-shaped structure consisting of two identical polypeptide heavy chains of molecular weight approximately 50 kDa each and two identical light chains (approximate molecular weight of 25 kDa each) linked by an S-S bond. The major parts of a mAb comprise (i) two identical Fabs (fragment-antigen-binding site), which form the arms of the Y, and (ii) an Fc (fragment crystallizable), which forms the stem of the Y. The constant region is responsible for triggering effector functions that eliminate the antigen-associated cells, and it must be tailored to match the requirements of the antibody to bind with a specific antigen [16]. The glycoproteins known as antibodies (Abs) are made by plasma cells and have the ability to attach to a particular antigen. They typically consist of two domains: a constant domain (Fc) that contacts immune cell receptors and a variable antigen-binding domain (Fab). The terms “cytotoxic molecules” and “warheads” refer to the payloads that significantly impact the characteristics and functions of ADCs. The harmful effects and effectiveness of ADCs are determined mainly by the mechanisms of cytotoxic chemicals. The linker bridges the antibody and payload and is the third crucial part of ADCs. To maintain the cytotoxic payload attached to the antibody, the linker must remain stable in blood; nevertheless, as soon as the ADC penetrates the tumor cell or is transferred to lysosomes, the linker must rapidly degrade to release the payload [17]. ADCs work together to target cancer cells in a “specific” way and kill them “efficiently.” These medications have the precision of a “biological missile,” capable of precisely eliminating cancer cells while expanding the therapeutic window and decreasing unintended side effects. ADC is endocytosed or internalized by cells to produce an early endosome, which is then followed by a maturation into late endosomes and, ultimately, a fusion with lysosomes once the mAb of ADC is bound to the target antigens that are particularly expressed on the cancer cells. The cytotoxic payloads eventually target DNA or microtubules and cause cell apoptosis or death by chemical or enzyme-mediated release in the lysosomes. The release of a permeable or transmembrane payload can potentially increase the effectiveness of ADC by inducing a bystander effect. Furthermore, these medications’ bystander effect might change the tumor microenvironment, increasing the efficacy of ADCs by killing more cells [18]. ADCs have been utilized in oncology to target cancer cells that express certain surface antigens, like HER2 and Trop-2, with strong cytotoxic drugs. With this focused strategy, the systemic toxicity of conventional chemotherapy is reduced, and efficacy is increased. ADCs have a multifaceted mode of action with a complicated mechanism (Figure 1). A cleavable or non-cleavable linker, or sometimes without any linker, a monoclonal antibody (mAb), and a cytotoxic payload comprise the three primary parts of the ADC. ADC searches for one particular antigen that is overexpressed on the surface of cancer cells as it is designed specifically to recognize and bind to it. When the ADC finds the target antigen and binds to it, the whole ADC complex is internalized via receptor-mediated endocytosis (Figure 1). Pinocytosis is a more universal mechanism than receptor-mediated endocytosis, as it is highly selective and needs certain receptors for internalization [19].

## 4. Antibody–Drug Conjugates in Treating Breast Cancer

**Figure 2:**
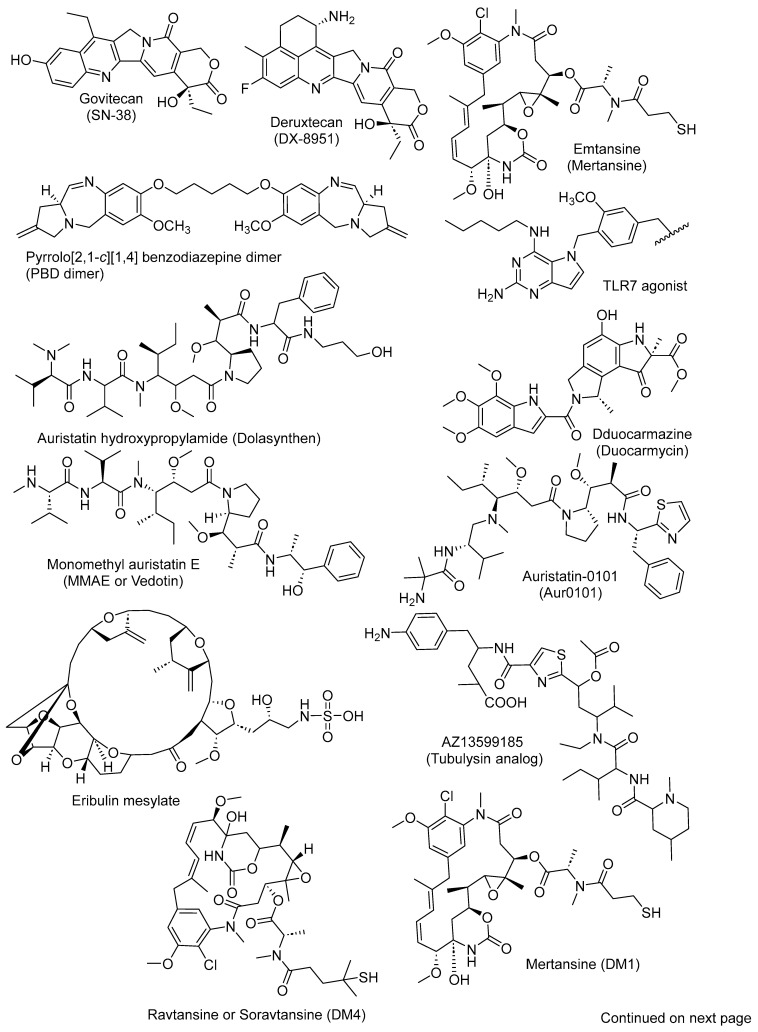

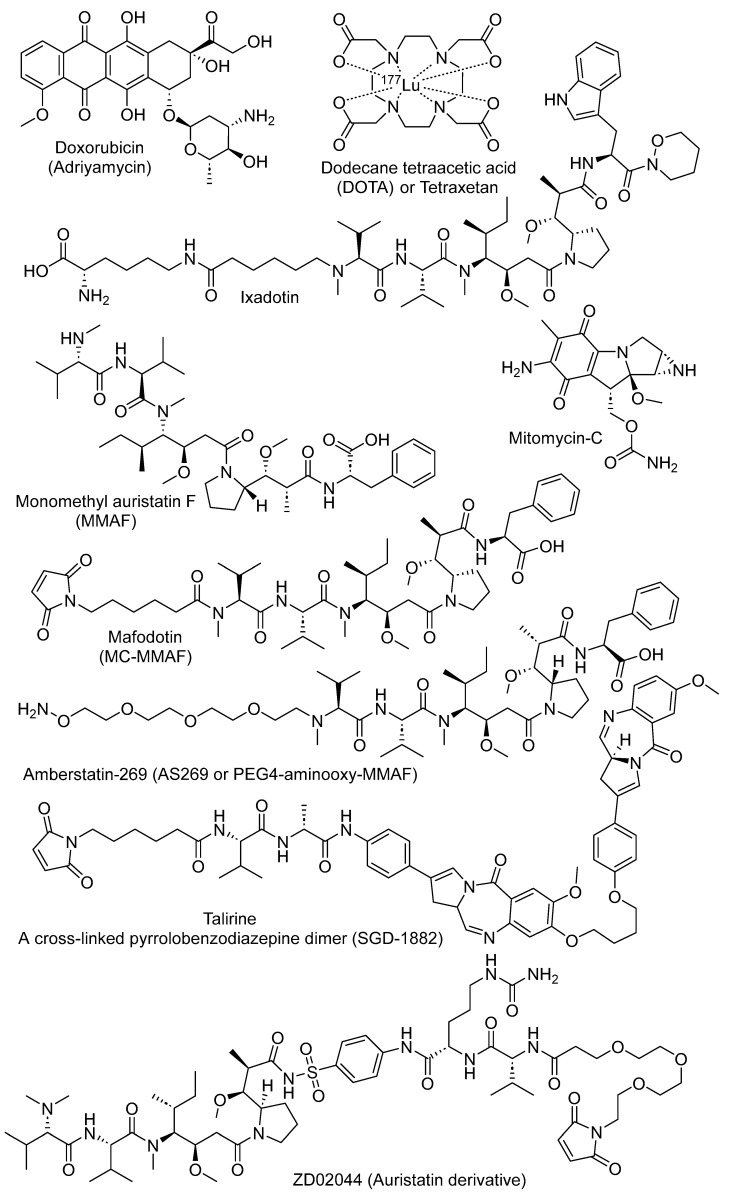
Chemical structures of the drugs (payloads) used to prepare anti-breast cancer ADCs (Table 1).

To date, 3 ADCs have been approved for therapeutic application in humans, and 26 others are in different stages of clinical trials. The constituent antibody and drug used to prepare a particular ADC, their therapeutic targets, and clinical trial phases are summarized in Table 1. The chemical structures of the drugs (payloads) used to prepare the ADCs are shown in Figure 2.

### 4.1. Approved ADCs Targeting Human Epidermal Growth Factor 2 (HER2)

At present, there are three approved ADCs for the treatment of HER2-positive breast cancer, and these are trastuzumab-emtansine (T-DM1), trastuzumab-deruxtecan (TDXd), and sacituzumab-govitecan (Table 1). The former carries a microtubule inhibitor (DM1) as payload, while the latter two carry topoisomerase I inhibitors deruxtecan and govitecan, respectively. Both were first introduced as anti-HER2 medications for HER2-positive breast cancer. These two HER2-targeting ADCs have increased overall survival rates with manageable side effects in the second- and third-line scenarios. In clinical research, several HER2-targeting ADCs that use cutting-edge technological developments in the conceptualization of the antibody, linker, and/or payload have demonstrated promising activity. Some of these are currently being assessed in more extensive clinical trials [104].

#### 4.1.1. Trastuzumab-Emtansine

T-DM1, also known as trastuzumab-emtansine, is an FDA-approved ADC for the treatment of HER2+ breast cancer [20]. The major target of T-DM1 is HER2 by using an mAb trastuzumab, which is conjugated to an anti-microtubule derivative by a stable linker (MCC). Conjugating DM1 with trastuzumab increases selectivity and may improve DM1 transport to cancerous cells. This unique treatment strategy can be used for HER2-positive cancer [21]. The first ADC to employ a thioether linker is T-DM1, which proved more stable in preclinical testing than other widely utilized linkers due to the presence of meager amounts of free DM1 in patient plasma, indicating its stability, which is probably why T-DM1 has a favorable safety profile [22]. T-DM1 offers better efficiency and a more favorable low-risk profile than capecitabine plus lapatinib, and T-DM1 is authorized to be used in individuals with trastuzumab plus a taxane-based prior therapy for metastatic HER2-positive breast cancer [23]. Reduced platelet count (5.7% of patients) and hypertension (2.0%) are the two most adverse events of grade 3 or higher in the T-DM1 group and radiation-related skin injury (1.0%) and hypertension (1.2%) in the trastuzumab group. A total of 94 patients (12.7%) who received T-DM1 and 58 patients (8.1%) who underwent trastuzumab medication experienced severe adverse effects. In the T-DM1 group, adverse events resulted in 133 patients (18.0%) stopping the study treatment, while in the trastuzumab group, 15 patients (2.1%) experienced the same outcome [23]. T-DM1’s dose-limiting toxicity is thrombocytopenia (TCP), a physical condition when the platelet count in the blood becomes too low [24]. A realistic example of trastuzumab-emtansine is SHR-A1201, which uses a chemical connector (linker) for integrating the chemotherapeutic medication DM1 with trastuzumab. A single dose of SHR-A1201 (1.2 mg/kg, 2.4 mg/kg, 3.6 mg/kg, or 4.8 mg/kg) was administered to patients using a conventional 3 + 3 dose-escalation methodology and 21 days were allotted to observe the dose-limiting toxicity (DLT). SHR-A1201 was administered to 12 patients, and elevated aspartate aminotransferase (75%), thrombocytopenia (75%), and nausea (66.7%) were the most often reported treatment-emergent adverse events (TEAEs), with most of them being grade 1 or 2 [25]. SHR-A1201 displayed the pharmacokinetic traits of standard ADCs. The 4.8 mg/kg dosage group showed a positive anti-tumor effect. When used in patients with advanced HER2-positive breast cancer, SHR-A1201 was safe and well tolerated. The immunogenicity data were in accordance with the clinical demands, and the pharmacokinetics measures displayed a linear trajectory [25]. A de-escalated study of trastuzumab-emtansine with endocrine therapy showed that tumors with de-escalated anti-HER2 treatment had an excellent prognosis when they had strong immune marker expression, were PIK3CA wild type and were luminal-A tumors (by PAM50) [26]. For patients with persistent invasive disease after neoadjuvant chemotherapy and HER2-targeted treatment, the phase 3 KATHERINE trial showed a substantial improvement in invasive disease-free life with adjuvant T-DM1 compared with trastuzumab. This research showed that GHS and functioning were mainly sustained in both treatment arms throughout time, irrespective of the variation in adverse outcomes. In both arms, mean ratings for patient-reported treatment-related symptoms indicated a little decline from baseline; however, these declines fell below the clinically significant criteria [27]. Since no methyl substitutions are next to the disulfide bond in a trastuzumab-DM1 conjugate prepared with the SPDP linker, it is the most minimally affected disulfide-containing design. The cytotoxic agent was intended to be released intracellularly by the disulfide-linked DM1-containing ADC upon endosomal reduction in the disulfide. Furthermore, DM1 generated from ADCs with a disulfide linker can be unleashed by antigen-expressing tumor cells, starting a “bystander” killing effect [28].

#### 4.1.2. Trastuzumab-Deruxtecan

Trastuzumab-deruxtecan, also known as DS-8201, is an antibody–drug conjugate that comprises a monoclonal antibody against HER2+ breast cancer. This antibody is conjugated to a cytotoxic inhibitor payload targeted to topoisomerase I. A tetrapeptide-based cleavable linker carries out this conjugation and has a DAR (drug-to-antibody ratio) of 8:1. DS-8201 is also effective against tumors with a low level of HER2 expression and can also target adjacent tumor cells that show expression of HER2 via bystander effect [29]. In a phase 1 study NCT02564900, trastuzumab-deruxtecan exhibited a bystander-killing effect in both HER2+ and HER2-low tumor models [30]. In the DESTINY—Breast04 phase 3 clinical trial—NCT 03734029, it was observed that the risk of disease progression was 50% lower in patients treated with trastuzumab-deruxtecan than with patients treated with chemotherapy of choice and also the fatality rate was reduced by 36% in the DS-8201 group of patients. In this trial, HER2-low status of the tumor was identified using IHC testing [29].

In the clinical trial NCT02564900, trastuzumab-deruxtecan was administered intravenously to participants at initial doses ranging from 0·8 to 8·0 mg/kg. Throughout 21 days, dose-limiting toxicities were evaluated, and dose reductions were applied as necessary. Patients underwent medication once every three weeks until their disease worsened or they experienced undesirable toxic effects. The primary outcomes were the determination of safety and the highest dose they could withstand. Trastuzumab-deruxtecan showed an anti-tumor effect in small, extensively pretreated research groups, even in tumors with modest levels of HER2 expression. Based on activity and safety, the most credible phase 2 dosage is 5·4 or 6·4 mg/kg [30]. The prescribed dosage of trastuzumab-deruxtecan (5.4 mg/kg of body weight), in clinical trial NCT03248492184, was administered to individuals who had an average of six prior treatments. Per the intention-to-treat analysis, 112 patients (60.9%; 95% confidence interval (CI), 53.4 to 68.0) showed improvement after receiving therapy. A follow-up of 11.1 months was the median (0.7–19.9) length of time. A median response length of 14.8 months (95% CI: 13.8 to 16.9) and a median progression-free survival period of 16.4 months (95% CI, 12.7 to not obtained) were recorded. The most frequent adverse events of grade 3 or above that occurred during the study were anemia (8.7%), nausea (7.6%), and a reduced neutrophil count (20.7% of the participants). According to another clinical evaluation, 13.6% of the patients (grades 1 or 2, 10.9%, grade 3 or 4, 0.5%, and grade 5, 2.2%) had interstitial lung disease concerning the trial medication [31]. Patients with HER2-positive metastatic breast cancer who were uncontrollable or resistant to trastuzumab-emtansine demonstrated strong efficacy in the single-arm DESTINY-Breast01 study when treated with trastuzumab-deruxtecan. Compared to DESTINY-Breast01, DESTINY-Breast02 demonstrates the favorable benefit–risk profile of trastuzumab-deruxtecan in patients with HER2-positive metastatic breast cancer. This was the first randomly assigned study to demonstrate the fact that resistance to a prior antibody–drug conjugate can be overcome by another one. The study is filed under the number NCT03523585 clinical trial [32]. The structure of T-DXd aimed to prevent off-target damage in healthy cells while delivering a strong cytotoxic payload to tumor cells harboring HER2. The new peptide-based linker was anticipated to be broken explicitly by lysosomal enzymes like cathepsins, which are elevated in the tumor microenvironment and persistent in plasma. Compared to T-DM1 (DAR 3–4), T-DXd has a drug-to-antibody ratio (DAR) of approximately 8 with homogenous conjugation, which is 2-fold higher. This allows for effective payload (cytotoxin) delivery to the intended cells. Since the ejected payload is membrane permeable, it can cause cytotoxicity in nearby tumor cells that are in close contact with the targeted cell, irrespective of the tumor cells’ extent of expression of HER2 (a cytotoxic bystander effect) [33]. In an open-label TUXEDO-1 study (NCT04752059), transtuzumab-deruxtecan has shown a high intracranial response rate in patients who have active brain metastases from HER2-positive breast cancer, and it is worth considering as a therapy alternative in this context [34]. Patients with metastatic/inoperable HER2-low breast cancer experience effective results with trastuzumab-deruxtecan (T-DXd), according to the results of the phase III DESTINY-Breast04 trial. DESTINY-Breast04 included 557 individuals with HER2-low tumors; most of these patients suffered from endocrine-refractory cancer that was HR positive. Patients were randomized 2:1 to receive T-DXd or the chemotherapy regimen recommended by their physician, which might include paclitaxel, capecitabine, or eribulin. The control group’s median progression-free survival (PFS) increased by two, from 5.1 months to 9.9 months. Additionally, the median overall survival (OS) increased from 16.8 months with chemotherapy to 23.4 months [35]. In HER2-negative patients, T-DXd was the initial HER2-targeted medication to show encouraging clinical anti-tumor effects with a tolerable risk profile [36,37]. Individuals who previously received pertuzumab are of major clinical interest, as recommendations indicate that conventional care for the initial treatment of metastatic HER2-positive breast cancer is simultaneous HER2-blockade with trastuzumab and pertuzumab along with chemotherapy [38]. Based on a combined total of 234 patients with HER2-positive breast cancer who received at least one dose of T-DXd at 5.4 mg/kg in trials DESTINY-Breast01 (184 participants) and DS8201-A-J101 (50 participants), the safety assessment for T-DXd was conducted. The treatment lasted a median of 7 months, ranging from 0.7 to 31 months. Trial DS8201-J101 enrolled 51 participants with HER2-positive breast cancer; however, one was not dosed. As a result, 50 patients were considered in the safety analysis of this trial. Patients who received T-DXd had severe, potentially deadly, or life-threatening interstitial lung disease (ILD), including pneumonitis. A total of 9.4% of the 234 patients with HER2-positive breast cancer who received 5.4 mg/kg experienced ILD, which the unbiased ILD committee determined was due to the medication [39]. In patients with HER2-positive breast cancer who had received extensive pretreatment, trastuzumab-deruxtecan demonstrated some initial efficacy and a controllable safety profile [40]. In the open-label, single-arm, phase II DESTINY-Breast01 study, patients with HER2-positive mBC resistant or refractory to T-DM1 received T-DXd 5.4 mg/kg intravenously every three weeks until disease progression, intolerable side effects, or withdrawal of consent. The main purpose of the study was to confirm the objective response rate (ORR) by independent central review (ICR). Overall survival (OS), progression-free survival (PFS), duration of response (DoR), and safety were among the secondary objectives [41]. The safety record of the patients who received DS-8201a was considered acceptable. The gastrointestinal and hematological systems are mostly associated with the most frequent adverse events (AEs). The incidence of nausea, vomiting, decreased appetite, anemia, alopecia, decreased neutrophil count, tiredness, and diarrhea exceeded 30% across all grades. High potency DS-8201a is for malignancies that are HER2 positive. A significant number of patients demonstrated a favorable response to the drug DS-8201a (ORR 37–79.9%) in the analyzed research, demonstrating the treatment’s effectiveness for patients with HER2-positive breast and gastric cancer. DS-8201a is more effective than other HER2-targeted medications such as margetuximab, neratinib, trastuzumab-emtansine, and lapatinib [42]. T-DXd was preferred by patients in a phase 3 clinical trial NCT03523585 compared to physician-recommended treatment. This indicates that, even with an extended treatment time, T-DXd did not adversely impact patients’ health-related quality of life. These findings confirm the overall advantage of T-DXd DESTINY-Breast02 with high potency and safety for patients with HER2-positive unresectable or metastatic breast cancer who had been treated with trastuzumab-emtansine previously [43]. A phase III randomized, multicenter, open-label study comparing trastuzumab-emtansine (T-DM1) and T-DXd was conducted by DESTINY-Breast03. Individuals with initial brain metastases (BMs) who had HER2-positive metastatic BC and whose disease condition worsened after receiving trastuzumab and a taxane benefited significantly from treatment with T-DXd as opposed to T-DM1 [44]. A significant anti-tumor effect was also detected in a small subset of individuals whose tumors did not express HER2, suggesting alternative mechanisms of action; HER2 is a predictor of sensitivity to T-DXd. The tumor microenvironment changes, HER2 expression reduction, and modifications to the cytotoxic effect of DXd are possible mechanisms of resistance to T-DXd. These findings suggest that in order to maximize treatment following T-DXd resistance, precise medical techniques based on molecular analysis are needed [45]. Based on a base case study, it was seen that the total expense of T-DXd is USD 1,266,945, while the value of T-DM1 is USD 820,082. Trastuzumab-deruxtecan’s total quality-adjusted life years (QALYs) was 5.09, while T-DM1 was 3.15. QALY is a measure that combines both quantity and quality of life—1 QALY is equal to one year of life in perfect health. The base-case incremental efficiency ratio (ICER) is the additional cost required to gain one more QALY with one treatment compared to another. In this case, it costs an additional USD 230,285 to gain one more QALY with trastuzumab-deruxtecan compared to trastuzumab-emtansine [46]. Various clinical trial phases of T-DM1 and T-DXd are summarized in Table 2.

However, as indicated before, resistance against DS-8201a may occur, and the resistance mechanism can broadly be categorized as (i) intrinsic resistance and (ii) acquired resistance. Intrinsic resistance to DS-8201a may happen due to alteration of *HER2* expression, *HER2* gene mutation, or activation of alternative signaling pathways. Alternatively, acquired resistance can be observed after the initial response to the DS-8201a treatment. The possible reasons might be the development of tumor heterogenicity, bypassing signaling pathways, *HER2* mutation, etc. The potential treatment strategies for recurrent/refractory BC patients experiencing DS-8201-resistance may involve (i) targeted therapies altering the payload, such as tucatinib or neratinib, which target HER2 through different mechanisms and may be beneficial; (ii) next-generation ADCs targeting HER2 or other significant antigens present in BC; (ii) analyzing genomic profile of tumors may help to determine potential treatment strategies; (iii) combination therapy that involves combining DS-8201a with other agents that inhibit downstream signaling pathways activated due to HER2 alterations—for example, adding PI3K inhibitors or mTOR inhibitors could be beneficial in patients whose tumors activate the PI3K/AKT/mTOR pathway. Alternatively, other small-molecule inhibitors that cause drug synergy with DS-8201a, such as taxanes and anthracyclines, can also be considered. (iv) Immunotherapy can play a significant role in the battle against DS-8201a-resistance BC. Checkpoint inhibitors such as pembrolizumab or nivolumab, which inhibit PD-1 in combination with other chemotherapeutic agents or targeted therapy in HER2-positive BC, deserve further investigation. In addition, therapeutic vaccines that evoke a strong immune response against tumor antigens could also offer a novel approach.

#### 4.1.3. Sacituzumab-Govitecan

Patients with metastatic triple-negative breast cancer, characterized by the absence of HER2, progesterone, and estrogen receptor expression in tumor cells, have a low prognosis. For formerly treated (beyond first-line) metastatic triple-negative breast cancer, single-agent chemotherapy is still the norm despite immunotherapy’s encouraging first-line clinical performance. Chemotherapy, however, has a poor rate of response and a very brief progression-free survival rate [47]. SN-38, the active metabolite of irinotecan, and a topoisomerase I inhibitor are linked to an anti-trophoblast cell surface antigen 2 (Trop-2) IgG1 kappa antibody via a patented hydrolyzable linker to form sacituzumab-govitecan, an antibody–drug conjugate. Over 90% of breast cancer cases exhibit the transmembrane calcium signal transducer Trop-2, which is highly expressed in a variety of tumor types. Targeted delivery of SN-38 to tumor cells is made possible by the anti-Trop-2 monoclonal antibody’s binding to Trop-2 expressed on the surface of tumor cells after treatment. Due to its membrane permeability, free SN-38 can either unleash intracellular SN-38 upon internalization or exert anti-tumor activity in nearby tumor cells (bystander effect) before the antibody–drug conjugate by linker dissolution. Sacituzumab-govitecan is a Trop-2-directed antibody–drug conjugate, which significantly outperformed chemotherapy in the phase 3 randomized clinical trial for patients with metastatic triple-negative breast cancer in terms of progression-free survival (hazard ratio for death or disease progression, 0.41; *p* < 0.001) and overall survival (hazard ratio for death, 0.48; *p* < 0.001). All clinical and predefined subgroups, including those previously receiving medication with PD-1 or PD-L1 inhibitors, benefited from sacituzumab-govitecan [47]. The transmembrane calcium signal transducer Trop-2 is highly expressed in metastatic breast cancer and has a significant effect on tumor growth and progression. Numerous intracellular signaling pathways, including NF-κB, Raf, and MAPK, have been linked to trop-2. Trop-2 activation was found in a broad spectrum of breast cancer subgroups in an immunohistochemical examination of 702 sequential breast cancer samples. HER2+ metastasis breast cancer (MBC) and other breast cancer subtypes have not demonstrated as much Trop-2 expression as HR+/HER2- breast cancer. Increased aggressiveness in breast malignancies and a poor prognosis for patient survival outcomes (such as OS and disease-free survival) have been linked to overexpression of Trop-2. When combined, Trop-2 is a possible new target for treatment and a prognostic indicator for patients who have advanced HR+/HER2- metastatic breast cancer [48]. TNBC is significantly more common in young patients, especially African American women who are in their menstruating phase. TNBC also frequently exhibits high levels of genomic instability and homologous recombination deficiency, which may indicate a patient’s susceptibility to platinum and DNA repair inhibitors like poly(ADP-ribose) polymerase-1 inhibitors. Advancement of targeted specific medicines enhances the management of TNBC, as most currently available drugs only achieve a progression-free survival (PFS) of less than 3.5 months. Consequently, we assessed sacituzumab-govitecan (IMMU-132), an antibody–drug conjugate (ADC) that targets Trop-2, a glycoprotein that is increased in several solid tumors, particularly TNBC [49].

##### Trophoblast Cell Surface Antigen 2 (Trop-2)

The 46 kDa glycoprotein known as Trop-2 was first discovered in a trophoblast cancer cell line and is frequently overexpressed in malignancies of the epithelium. Trop-2 has several different biological functions, one of which is the transduction of cytoplasmic Ca^2+^, which is reliant on a particular phosphorylation site on protein kinase C. Trop-2 possesses carcinogenic qualities, as does the bicistronic CYCLIN D1-Trop-2 mRNA chimera. An unfavorable prognosis is associated with Trop-2 overexpression in a number of malignancies, including breast cancer [50].

##### Impact of Sacitizumab-Govitecan

The transmembrane glycoprotein (sacituzumab), which plays a role in transmitting calcium signals, has both intracellular and extracellular elements. For multiple reasons, sacituzumab-govitecan (SG) perfectly fits as an ADC. First, its membrane-permeable active metabolite, SN-38, is thought to be 2–3 fold more effective than irinotecan and can have a “bystander effect.” Secondly, SG’s hydrolyzable linker provides an additional mechanism for the “bystander effect” by enabling the extracellular release of SN-38 in addition to its intracellular release. Tumors with heterogeneous Trop-2 expression may benefit most from this extracellular release potential. SG provides a high DAR of 7.6:1. Previous ADCs, including T-DM1, had DARs of 4:1 or less; however, SG can retain a larger DAR without sacrificing pharmacokinetic or antibody binding characteristics because of the unique design of the antibody and linker. In conclusion, sacituzumab-govitecan was found to be less harmful when compared to other topoisomerase inhibitors, especially when it comes to milder diarrhea. This is thought to be because, in contrast to SN-38, metabolized straight from irinotecan, SN-38 molecules attached to antibodies have a reduced rate of glucuronidation [51]. The active ingredient in irinotecan (CPT-11), a topoisomerase I inhibitor, is camptothecin, which is known as SN-38, an anticancer drug. In the clinical trial NCT01631552, the maximum tolerated dose, as measured by tolerance to a single cycle of therapy, was found to be 12 mg/kg after doses of 8, 10, 12, and 18 mg/kg were investigated. Doses had to be lowered because of regular delays during or between cycles at this dosage. Therefore, enrollment was kept at two lower dose levels, 8 and 10 mg/kg, to ensure patients received numerous medication cycles with little interruptions or dose decreases. CTCAE, version 4.03, was used to evaluate safety, where neutropenia was observed. To prevent further neutropenia, researchers were allowed to administer a granulocyte-colony-stimulating factor cytokine after the initial dosage during the phase 2 expansion part of the study. A total of 18 patients in the 8 mg/kg group (22%) and 25 patients in the 10 mg/kg group (26%), respectively, had at least one hematologic cytokine support medication at the time of the study. In the 8 mg/kg cohort, seven patients (8.6%) experienced an adverse event at the end of the study, two of which were thought to be connected to the medication. In the 10 mg/kg group, ten patients (10.3%) experienced an adverse event at the end of the trial, seven of which were thought to be connected to the medication [52]. IMMU-132 contains 7.6 SN-38 molecules/IgG on average. SN-38 is a reasonably toxic drug that is utilized therapeutically in the form of irinotecan. Most other ADCs with stronger medication have a replacement level of approximately three or four molecules/IgG.14 The increased SN-38 replacement level probably makes up for SN-38’s less potent potency, but it has no discernible effect on the PK behavior of IMMU-13214, 18. On the other hand, the clinical trial results show that toxicity has been controlled in a wide range of individuals, with safety and efficacy evaluations suggesting that a beginning dose of 10 mg/kg is appropriate. Like most other ADCs, IMMU-132 employs an internalizing antibody, which is thought to serve as a crucial component of an effective ADC. Still, in contrast to other conjugates that need a highly stable linker, the linker used in IMMU-132 permits SN-38 to release slowly because preclinical research revealed that this kind of linker was more appropriate for SN-38 than a more stable type [52]. It has been demonstrated that topoisomerase I inhibitors, such as SN-38, the payload of SG, promote double-stranded DNA breaks independent of the presence of BRCA mutations. In translational models of TNBC with a BRCA mutation, tumor growth decreases and may give TNBC tumors synthetic lethality. SG became the first Trop-2-directed antibody–drug conjugate to show a significant PFS and OS benefit when compared to standard-of-care chemotherapy in the second-line or greater mTNBC setting in the clinical trial (NCT02574455) phase III ASCENT study. SG also showed better results when compared to TPC (eribulin, vinorelbine, gemcitabine, or capecitabine). However, the patient cohort that generated these efficacy findings was not chosen based on Trop-2 expression. The efficacy outcomes in this prespecified biomarker study are evaluated based on the expression of Trop-2 on the tumor membrane and the presence of BRCA1/2 mutations. According to the results, the SG arm outperformed the TPC arm in terms of efficacy in both the high and medium Trop-2 expression categories. Both BRCA1/2-positive and -negative patients benefited from SG more than TPC. The main research limitations of this work are that this sub-analysis did not include formal testing between treatment arms, and ASCENT lacked the power to identify predictive effects [53]. In the ASCENT trial, TNBC was not present at the time of the first breast cancer diagnosis for almost one-third of the patients. Nonetheless, those lacking TNBC at first diagnosis saw a similar clinical benefit from SG over TPC as did patients with TNBC at first diagnosis and the entire ASCENT primary analysis cohort [54]. The sacituzumab-govitecan anti-Trop-2-SN-38 conjugate demonstrated an urging therapeutic index in a clinical trial NCT01631552, in spite of using a more traditional drug that is not thought to be ultra toxic (drugs active in the picomolar range, whereas SN-38 has potency in the low nanomolar range). The conjugate was clinically active in a variety of solid cancers at doses with mild and feasible toxic effects [55]. A novel drug-release pattern is attributed to SG’s hydrolyzable linker, which exhibits a dual mode of action by cytotoxic action after internalization and tumor kill via a bystander effect. SG also has a high DAR and uses camptothecin, which is considerably more toxic than its prodrug, irinotecan [56]. With no documented cases of severe peripheral neuropathy, interstitial lung disease, or cardiac toxicity, SG has a controllable safety profile. ASCENT’s adverse event (AE) data show that SG’s safety profile is mostly controllable. However, SG had a larger percentage of patients with specific adverse events (AEs) than TPC, such as grade 3/4 neutropenia and diarrhea. It is worth mentioning that adverse events can lower the quality of life (QoL). SG has received FDA approval for patients with unresectable locally advanced tumor necrosis or metastatic tumor necrosis and who have had ≥2 systemic treatments, including ≥1 for metastatic illness, consistent with earlier research [57]. The clinical advantage of SG over single-agent chemotherapy in PFS (progression-free survival) and OS (overall survival) has been validated by the subsequent evaluation of the ASCENT trial, which included patients both with and without baseline brain metastases. SG efficacy was established across Trop-2 expression subgroups. SG significantly improved outcomes for patients with the two most Trop-2-expressing quarters in PFS, OS, and ORR [58]. A population pharmacokinetics study (PopPK) used data from two pivotal studies in patients with metastatic triple-negative breast cancer (mTNBC) and other solid tumors to characterize the pharmacokinetics of SG and its constituents completely. For SG and its payload, tAB, separate two-compartment PopPK models were created; for free SN-38, a sequential method was used based on parameters inferred from the SG model. The clearance and volume parameters were scaled using body weight as the basis, and the impact of important clinical variables was evaluated. Goodness-of-fit plots showed no discernible bias in the models’ precision, and population predictive checks validated the models’ accuracy in projecting the observed data. Taken together, these studies supported the practical use of SG. They informed effective dosage regimens in patients with advanced solid tumors, especially in mTNBC, by thoroughly understanding the drug’s pharmacokinetics, free SN-38 release kinetics, and tAB disposition [59]. On 22 April 2020, the FDA expeditiously approved SG, marking the drug’s first approval globally. Furthermore, it is the first ADC the FDA has expressly approved for mTNBC. Particularly for those who are substantially pretreated, as was the case with patients involved in the IMMU-132-01 trial, patients with mTNBC have few therapy options and urgently require new, safer, and effective medicines. Sacituzumab-govitecan showed a good benefit–risk profile in the IMMU-132-01 study, with an enhanced ORR and DoR compared to other treatments. However, SG treatment was related to many common side effects, including nausea, neutropenia, diarrhea, fatigue, anemia, vomiting, constipation, rash, decreased appetite, and stomach discomfort. These adverse effects could be tolerated when supportive measures, dose changes, and monitoring were implemented [60]. A similar ADC, datopotamab-deruxtecan, which targets Trop2 in TNBC treatment and has the potential to replace sacituzumab-govitecan has been discussed later in this review. However, despite the promising clinical outcomes of SG, questions remain unsolved regarding its efficacy, safety profile, and Trop-2 biological role in cancer. Trop-2 should not be designated as a predictive biomarker in SG treatment, albeit its expression correlates with disease outcome, yet its levels are not uniform across all TNBCs. This has prompted interest in searching for non-Trop2 ADCs that may address these challenges while offering further treatment strategies. By targeting different antigens and following diverse mechanisms, non-Trop2 ADCs have the potential to improve therapeutic outcomes with reduced side effects. Although non-Trop2 ADCs may offer additional benefits, which include the possibility of overcoming drug resistance, and reduced toxicity through different mechanisms of action and/or in combination with other therapeutic agents (combination therapy), the development of non-Trop2 ADCs in the treatment of TNBC is still in its early stage. In-depth research and extensive clinical trials are required to determine the exact role(s) of non-Trop2 ADCs in the treatment of TNBC.

## 5. Investigational Anti-Breast Cancer ADCs

### 5.1. Patritumab-Deruxtecan

Human epidermal growth factor receptor 3 expression can be seen in a variety of breast cancer and other solid tumors. To date, no ADC has received approval for HER3-directed treatment. Patritumab-deruxtecan is a new-generation HER3-directed antibody–drug conjugate (U3-1402) that constitutes a immunoglobulin G1 mAb patritumab linked to a topoisomerase I Inhibitor cytotoxic payload by a tetrapeptide, cleavable linker, which is specific to a particular tumor. Clinical trial NCT02980341 was an open-label study including patients with breast cancer expressing HER3 to determine the maximum tolerated dose along with the safety and efficiency of patritumab-deruxtecan. Patients were treated with doses ranging from 1.6, 3.2, 4.8, 6.4,or 8.0 mg/kg once every three weeks. In the initial phase of the study, patients received a dose of 3.2 mg/kg, followed by 4.8 mg/kg in the second phase, and 6.4 mg/kg in the third and subsequent phases. Another dosing approach involved administering 4.2 mg/kg every two weeks for three cycles, followed by 6.4 mg/kg every three weeks. The primary goals of the study were to determine the recommended dosage for expansion (RDE) and to assess the safety and efficacy of the different dosing schedules. Based on the results, the fixed-dose regimens of 4.8 mg/kg and 6.4 mg/kg administered every three weeks were selected for further evaluation in terms of dose expansion, safety, and efficacy, while other dosing patterns were not pursued further. The dose-escalation and dose-finding phases included patients with any clinical subtype of HER3-high-expressing breast cancer. The dose-expansion group included patients with HER3-high or HER3-low-expressing HR+/HER2- breast cancer, as well as those with HER3-high-expressing triple-negative breast cancer (TNBC) [61]. Despite a small sample population, HER3-DXd demonstrated anticancer activity in HER2+ breast cancer patients (n = 14), all of whom had experienced disease progression after previous anti-HER2 therapy. While HER3 expression is associated with resistance to HER2-targeted therapies, the findings suggest that HER3-DXd warrants further research as a potential treatment option following the progression of anti-HER2 therapies. Early in the treatment process, a reduction in the proportion of tumor cells expressing HER3 on the membrane was observed, possibly due to the treatment reducing the number of HER3-expressing tumor cells or the binding and internalization of the HER3/HER3-DXd complex. The strong and prolonged response in a heavily pretreated patient group indicates that HER3-DXd maintains anticancer activity, even though the reduction in HER3 expression may have diminished the drug’s effectiveness over time. The safety profile of HER3-DXd was generally acceptable, with only 9.9% of patients discontinuing treatment due to adverse events. The most common side effects were gastrointestinal and hematologic, which were typically managed through dose adjustments, with no significant cases of bleeding or infections [61].

### 5.2. DHES0815A-THIOMAB

Pyrrolobenzodiazepines, or PBDs, are strong cytotoxic substances that crosslink alkylate DNA. The PBD dimer is altered to alkylate DNA but not to crosslink it. In cynomolgus monkey safety investigations, this HER2 ADC, DHES0815A, ClinicalTrials.gov: NCT03451162, shows good tolerability and in vivo efficacy in HER2-positive and HER2-low tumor models. The onset of DNA damage and death, activity in non-dividing cells, and bystander activity are some of the mechanisms of action. The phase 1 trial was terminated early due to constant, non-resolvable cutaneous, ophthalmic, and pulmonary toxicity in patients at increased doses despite early signals of anti-tumor effectiveness. In DHES0815A, a humanized 7C2 (hu7C2) HER2 THIOMAB antibody is combined with a modified PBD (pyrrolobenzodiazepine) dimer, PBD-monoamide, via a disulfide linker attached at a cysteine designed into light chain site lysine 149 (LC K149C; Kabat and EU numbering) optimized for enhanced stability. This modification allows the antibody to bind to subdomain I of the HER2 ECD. DHES0815A exhibited anti-tumor efficaciousness in HER2+ models of gastric and breast cancer in non-clinical models, including animals that are unresponsive to T-DM1. The phase 1 dose-escalation study demonstrated early indications of clinical efficacy for DHES0815A. But as the patients’ treatment went on, some worrisome toxicities surfaced. It is possible that DNA damage accumulates in some organs at increased doses of DHES0815A, leading to toxicity signals. However, considering a longer recovery period in the cynomolgus monkey safety research, it is surprising that the character of these toxicities in patients was marked, as opposed to cynomolgus monkeys [62].

### 5.3. Datopotamab-Deruxtecan

Datopotamab-deruxtecan is a antibody–drug conjugate which is used to treat Trop2-expressing breast cancer. Datopotamab-deruxtecan is made of a mAb, which is targeted against Trop2 and is linked covalently to a cytotoxic payload topoisomerase I inhibitor by a tumor-specific 4 peptide-based linker which is known to be a derivative of exatecan. Datopotamab-deruxtecan is a highly potent targeted ADC which, when internalized into tumor cells expressing Trop2, results in neutralization of the target cell and also leads to killing of the cells in the nearby tumor microenvironment by the bystander-killing effect. In the TropION-PanTumor01 clinical trial (ClinicalTrials.gov NCT03401385), Dato-DXd was administered intravenously on day 1 of each 21-day cycle. Patients continued treatment until they no longer responded, their disease progressed (PD), or they withdrew consent. The study determined that a dose of 6 mg/kg every three weeks provided the optimal benefit–risk ratio, establishing this as the Recommended Dose Expansion (RDE). During the dose-expansion phase, patients received the RDE of 6 mg/kg every three weeks. The primary objective of the trial was to assess the safety and tolerability of Dato-DXd during dose escalation and expansion. Secondary objectives included evaluating the pharmacokinetics (PK) of Dato-DXd, the total anti-Trop2 antibody, and the DXd payload MAAA-1181a. Additionally, the study aimed to assess the anticancer activity of Dato-DXd and monitor the development of anti-drug antibodies (ADAs). The confirmed objective response rate (ORR) was 26.8% in patients with HR+/HER2- breast cancer and 31.8% in those with triple-negative breast cancer (TNBC), indicating clinical activity. The duration of response (DOR) was not estimable (NE) for HR+/HER2- breast cancer, and it was 16.8 months for TNBC, suggesting durable responses. Notably, the TNBC subgroup that had not been previously treated with topoisomerase I (topo I) inhibitors showed a higher ORR and longer median overall survival (OS) and progression-free survival (PFS) compared to the overall TNBC population, suggesting that prior exposure to topo I inhibitors may contribute to resistance. Dato-DXd’s selective delivery of its payload is enabled by a cleavable, plasma-stable linker that releases DXd through proteolytic degradation by lysosomal enzymes in tumor cells. This targeted approach reduces systemic exposure and leads to sustained therapeutic responses, thereby improving the drug’s benefit–risk profile. This might explain the relatively lower rates of neutropenia and diarrhea observed in the study compared to sacituzumab-govitecan (SG) [63].

### 5.4. Dolasynthen B7-H4 Directed ADC

Dolasynthen B7-H4 Directed ADC, also known as XMT-1660, is a novel optimized antibody–drug conjugate which targets the plasma membrane protein B7-H4(VTCN1). The plasma membrane protein B7-H4 (VTCN1) is expressed in ovarian, endometrial, and breast cancers, among other tumor types. Its expression in normal tissues is quite low. There have been reports of B7-H4 having an immunosuppressive role, and its expression in tumors has been linked to a decreased incidence of immune cells invading the tumor. The drug-to-antibody ratio of XMT-1660 is 6 and it showed good efficacy in a preclinical trial. In the preclinical phase, in vivo comparison showed that XMT-1660 completely stopped tumor progression and tumor size decreased. When it came to B7-H4, the site-specific dolasynthen (DS), with a drug-to-antibody ratio (DAR) of 6 ADC (XMT-1660), outperformed the DS DAR 2 and DF DAR 12 ADCs in terms of pharmacokinetics, toxicological profiles, and in vitro anti-tumor efficacy. Selecting the correct DAR is the main consideration when engineering an ADC. In both examined tumor models, XMT-1660 showed better anti-tumor activity than the other ADCs [64]. XMT-1660 is being studied in a phase I clinical trial (NCT05377996) where patients will receive an intravenous dose of XMT-1660 once every three weeks. The first objective of the study is to determine the safety and efficiency of XMT-1660 [65].

### 5.5. Trastuzumab-TLR 7 Agonist NJH395

TLR 7 agonist NJH395 is a novel ISAC (immune stimulator–antibody conjugate) including a non-cleavable linker payload that binds an anti-HER2 antibody to a Toll-like receptor 7 (TLR7) agonist. According to preclinical characterization, antigen targeting and TLR7 agonism contribute to anticancer efficacy, and ISAC-mediated activation of myeloid cells in the presence of antigen-expressing cancer cells is demonstrated. An immunoglobulin G1 (IgG1) antibody (MIW338) coupled to an immunostimulatory TLR7 agonist linker payload makes up NJH395, an ISAC. A humanized monoclonal antibody called MIW338 interacts with HER2. MIW338 has a sequence that is comparable to trastuzumab and is a member of the IgG1/κ isotype subclass. To improve stability, four cysteine residues were added for site-specific conjugation via a maleimide ring with the TLR7 agonist linker payload. The anti-HER2 antibody worked selectively to deliver the TLR7 agonist to HER2+ tumor cells. This was accompanied by payload uptake in APCs, which occurred either by phagocytosis or payload catabolite release in the TME. TLR7 was then activated, which set off a type I IFN response and selective immunomodulation of the tumor microenvironment (TME), which was marked by the activation of T cells and myeloid cell proliferation [66].

### 5.6. SGN-CD228A

An experimental antibody–drug conjugate (ADC) called SGN-CD228A is targeted at the cell surface protein known as melanotransferrin (CD228, MELTF, MFI2, p97), which was initially discovered in melanoma, but recent IHC studies discovered that SGD-CD228A is also expressed in NSCLC and triple-negative breast cancer (TNBC) as well. SGN-CD228A consists of microtubules disrupting monomethyl auristatin E (MMAE) linked to a monoclonal antibody, hL49. This SGN-CD228A has high specificity for CD228 melanotransferrin protein. The SGN-CD228A is linked to the mAb by a glucuronide linker. The cytotoxic effect of SGN-CD228A depends on CD228 expression, internalization, and the inherent sensitivity to the MMAE payload. MMAE, a synthetic analog of dolastatin-10, a natural microtubule-disrupting compound, was chemically linked to the antibody via a PEGylated glucuronide linker that featured a self-stabilizing maleimide. In a phase I clinical trial (NCT04042480), it was seen that CD228 expresses itself highly on tumor tissues and poorly on normal tissues, making it a suitable ADC target. In addition to its involvement in angiogenesis and metastasis, CD228 may potentially be inhibited by a treatment targeted at the protein. Taken together, the findings imply that using an ADC to target CD228 may be a viable tactic to maximize effectiveness by delivering a cytotoxic payload and blocking important elements of tumor biology [67].

### 5.7. Trastuzumab-Duocarmazine

SYD985, also known as trastuzumab-duocarmazine, is an antibody–drug conjugate which targets HER2 expression in breast cancer. SYD985is engineered by linking a mAb IgG1 trastuzumab antibody to a cytotoxic linker drug containing duocarmycin. SYD985 has a DAR of 2:8:1. In trastuzumab-duocarmazine, the linker–drug combination consists of a cleavable linker and the prodrug seco-duocarmycin–hydroxybenzamide–azaindole (seco-DUBA). Once trastuzumab binds to HER2 and is internalized, lysosomal proteases cleave the linker, releasing the active toxin (DUBA). This toxin causes DNA damage by alkylating it, leading to the death of both dividing and non-dividing cells. Additionally, proteases secreted by cancer cells, such as cathepsin B, can also cleave the linker extracellularly. The ongoing phase 3 TULIP trial (NCT03262935) is comparing trastuzumab-duocarmazine with standard chemotherapy combinations in patients with HER2-positive breast cancer. Furthermore, single-agent trastuzumab-duocarmazine has demonstrated efficacy in patients with HER2-low (IHC 1+ or IHC 2+ ISH-negative) hormone receptor-negative disease, a group for which no HER2-targeted therapies or antibody–drug conjugates are currently approved [68].

### 5.8. MORAb-202 (Farletuzumab)

MORAb-202 is a new antibody–drug combination whose payload is eribulin, a microtubule inhibitor, coupled to farletuzumab, an anti-folate receptor α antibody. In the tumor microenvironment, erybibulin exhibits special properties such as antimitotic activity, vascular remodeling, and reversal of the epithelial–mesenchymal transition. Different antibody–drug conjugates are anticipated to exhibit distinct safety and clinical effectiveness profiles. The mechanism of action of MORAb-202 differs from those of other antibody–drug conjugates. The active payload of MORAb-202 is released into target cells in lysosomal compartments by the unique cathepsin-B cleavable linker, which does not break down in serum. Consequently, compared to other antibody–drug conjugates, it might exhibit less severe systemic adverse effects. One of the four glycopolypeptide folate receptors (FRα, FRβ, FRη, and FRδ), which have molecular weights ranging from 38 to 45 kDa, is responsible for transporting folate. Solid tumors, such as lung, breast, and ovarian malignancies, overexpress the glycosylphosphatidylinositol-anchored membrane protein FRα. A synthetic analog of halichondrin B, eribulin mesylate (eribulin) suppresses microtubule dynamics and is licensed to treat metastatic breast cancer. A cathepsin-B cleavable linker connects farletuzumab to eribulin in MORAb-202, an antibody–drug combination. For an average drug-to-antibody ratio of 4.0, the link—a decreased interchain disulfide—binds to maleimido-PEG2-valine-citrulline-p-aminobenzylcarbamyl-eribulin. Patients with FRα-positive solid tumors enrolled in the first-in-human phase 1 study of MORAb-202 (NCT03386942) showed that MORAb-202 is well tolerated when given every three weeks at doses ranging from 0.3 to 1.2 mg/kg. These dosages result in eribulin at equivalent concentrations of 0.2 to 0.85 mg/m^2^. These are less than the maximum amount of eribulin mesylate permitted, which is 1.4 mg/m^2^ (or 1.23 mg/m^2^ eribulin) on days 1 and 8 of a 21-day cycle [69].

### 5.9. MEDI4276 (Derivative of Trastuzumab)

Featuring site-specific linkage to a tubulysin-based microtubule inhibitor payload, MEDI4276 is a biparatopic tetravalent antibody that targets two non-overlapping epitopes within subdomains 2 and 4 of the HER2 ecto-domain. In vitro, MEDI4276 exhibits improved HER2-positive tumor cell internalization and cytolysis. An experimental ADC called MEDI4276 consists of a biparatopic tetravalent mAb that attaches to two different HER2 epitopes. The primary antibody is a 39S-directed completely human (XenoMouse derived) antibody that targets subdomain 2 of the HER2 ECD. MEDI4276 can inhibit HER2/HER3 receptor phosphorylation in recombinant heregulin-β1-stimulated cancer cells, just like pertuzumab. To create MEDI4276, the amino terminus of the 39S IgG1 heavy chain was genetically linked to the scFv of trastuzumab, which binds HER2 ECD subdomain 4 with great affinity. MEDI4276 inhibits the heterodimerization of HER2:HER3 when heregulin β-1 is present. The location of the 39S and trastuzumab epitopes on the HER2 ECD is at the opposite ends, more than 90 Å apart, according to the co-crystal structure of the 39S Fab-HER2 complex. As a result, the *N*-terminal amino acid of the 39S heavy chain and the *C*-terminal residue of trastuzumab scFv cannot bind to the same HER2 receptor molecule at the same time. Instead, the biparatopic design causes receptor clustering at the cell surface by crosslinking nearby HER2 receptors. Rapid receptor internalization, recycling inhibition, and intracellular trafficking towards lysosomal degradation are the outcomes of this clustering. With a bystander-killing effect, the tubulysin warhead AZ13599185 is used in the antibody–drug combination MEDI4276, which targets both HER2-positive and HER2-negative tumor cells. A maleimidocaproyl linker is used to achieve site-specific conjugation to cysteines inserted at heavy chain residues 239 and 442 of the antibody, which results in this effect. The average DAR (drug-to-antibody ratio) after this combination is approximately 4. Compared to T-DM1, AZ13599185 has significant efficacy across a wide range of tumor cell types by inducing apoptotic cell death in tumor cells across a diverse tumor cell population by inhibiting microtubule polymerization during mitosis. MEDI4276 exhibited non-cross-resistance to T-DM1 and sustained objective clinical responses in the first-in-human phase 1 study, consistent with findings from other more recent HER2-targeted antibody–drug conjugates (ADCs). MEDI4276’s efficiency might be affected because it internalizes and traffics to lysosomes more quickly than T-DM1. Since these processes are usually evaluated on shorter laboratory timelines (minutes to hours), it is unknown how much they impact clinical outcomes over the course of weeks to months [71]. MEDI4276 has demonstrated clinical activity, especially in breast cancer, yet obstacles remain in the way of its continued development. A negative pharmacokinetic (PK) profile is one of them; it could not sufficiently overcome antigen sinks, non-targeted tissues expressing the same antigen, and sequestering the medicine from tumor cells. Moreover, MEDI4276 has a significant toxicity profile, which further restricts its therapeutic applicability. As a result, even though MEDI4276 shows promise in terms of effectiveness and non-cross-resistance, these issues must be resolved if the medication is to go through the clinical development phase [70] successfully.

### 5.10. Glembatumumab-Vedotin

ADC, comprised of glembatumumab with vedotin (also known as CDX-011 or CR011-vcMMAE), includes a potent microtubule inhibitor, monomethyl auristatin E (MMAE or vedotin), with a completely human IgG2 monoclonal antibody directed against the tumor-associated antigen glycoprotein NMB (gpNMB). The purpose of glembatumumab-vedotin is to attach gpNMB and, upon internalization, release MMAE by the breakdown of a valine-citrulline peptide linker that is susceptible to proteases, hence causing microtubule inhibition and tumor cell death. A type I transmembrane protein called gpNMB is expressed more frequently and widely in human cancerous tissues than in healthy tissues. In preclinical models, overexpression of gpNMB increases angiogenesis, inhibits tumor cell death, and encourages invasion and metastasis by a variety of tumor types. Overexpression of gpNMB in breast and small-cell lung cancer seems to be a poor prognostic indicator [71]. The gpNMB is expressed both intracellularly and on the surfaces of cells. A sheddase called ADAM10 can split surface gpNMB and produce a soluble, physiologically active gpNMB molecule [72].

Glenmabusumab-vedotin demonstrated encouraging efficacy in treating triple-negative breast cancer (TNBC) in the CR011-CLN-20 study and the ensuing EMERGE analysis, especially in patients whose tumors overexpress gpNMB (glycoprotein NMB). It is established that in TNBC, gpNMB correlates with the metastatic phenotype. With a criterion of ≥25% malignant epithelial cells expressing gpNMB, the EMERGE research identified a subpopulation of patients with gpNMB-overexpressing TNBC who may benefit from improvements in progression-free survival (PFS) and overall survival (OS). Because the expression of gpNMB was generally stable over time and this cutoff captured a considerable number (41%) of screened TNBC patients, using archival samples for eligibility determination was made possible. Nevertheless, the exploratory character of the analyses and the small sample sizes in some subgroups hampered the EMERGE analysis. To validate these results, the METRIC pivotal study has been started. In the METRIC trial, 300 women with metastatic gpNMB-overexpressing TNBC will be enrolled and randomly assigned to receive either capecitabine or glembatumumab-vedotin in a 2:1 ratio. PFS is the primary outcome of METRIC, and its secondary outcomes are safety evaluations, OS, objective response rate (ORR), duration of response, and quality of life metrics. By addressing the shortcomings of earlier studies and offering more substantial proof of glembatumumab-vedotin’s clinical efficacy and safety profile in this particular patient population, the trial aims to validate the possible benefits seen in gpNMB-overexpressing TNBC patients receiving treatment with the medication [71].

### 5.11. PF-06650808 (Cofetuzumab)

A new anti-Notch3 antibody–drug conjugate, PF-06650808, can inflict cytotoxicity on target cells by delivering an auristatin-based payload. Through the transcriptional activity of its intracellular domain, the highly conserved type I transmembrane glycoprotein Notch3 receptor controls cell survival, proliferation, and differentiation in several organs. In experimental animal models, constitutive stimulation of Notch3 signaling may be carcinogenic and induce tumors. It has been discovered that overexpressing activated Notch1 and Notch3 in transgenic mice prevents the development of the mammary gland and causes breast tumors. Moreover, some investigations have shown that malignant cells, such as those from breast, ovarian, and lung cancers, overexpress or amplify Notch3. In a phase I trial (NCT02129205), 40 patients with advanced breast cancer (BC) and other solid tumors unselected for Notch3 expression were studied for safety, pharmacokinetics, immunogenicity, and early anticancer efficacy of single-agent PF-06650808. Determining the dose-limiting toxicity (DLT) was the primary goal of the study. Every three weeks, PF-06650808 was injected intravenously at a beginning dose of 0.2 mg/kg. The modified continued reassessment approach increased the dose to 6.4 mg/kg. Patients with advanced ER+ breast cancer were tested at a higher dose level of 2.0 mg/kg. Nearly all patients (90%) had received ≥3 lines of anticancer therapy prior, and the majority of patients (60%) had advanced BC. In general, PF-06650808 treatment was well tolerated at doses ≤2.0 mg/kg without DLTs. An estimated 2.4 mg/kg was the maximum tolerated dosage (MTD). Fatigue (40.0%), decreased appetite (37.5%), nausea (35.0%), alopecia (32.5%), abdominal discomfort (25.0%), pruritus (25.0%), and vomiting (25.0%) were the most frequent adverse events (AEs) associated with treatment for all patients. Tumor size decreased in one patient group (Group 1) as the cumulative area under the curve (AUC) ADC values increased, i.e., higher drug exposure is associated with better tumor response (i.e., reduction in tumor size). AUCADC represents the total exposure of the drug over time, calculated as the area under the curve (AUC) of the drug concentration vs. time graph. Higher AUCADC indicates higher cumulative exposure to the drug. Group 2, on the other hand, did not exhibit a tumor response trend over the whole range of cumulative AUC ADC values, which means that the tumor response does not appear to be influenced by the level of drug exposure [73].

### 5.12. Trastuzumab-Auristatin (PF-06804103)

Anti-HER2 immunoglobulinG1 ADC, PF-06804103, is made up of an anti-HER2 monoclonal antibody (trastuzumab) coupled to the cytotoxic agent Aur0101 at specially designed reactive cysteine sites. This combination allows for an almost homogenous (drug–antibody ratio = 4) ADC synthesis. PF-06804103 acts by a minimum of two distinct processes. The main approach involves specifically targeting HER2-positive or HER2-low-expressing cells with the cytotoxic anti-microtubule auristatin payload. In cancer cells that express HER2, trastuzumab inhibits HER2-mediated signaling, which is an additional mode of action. Preclinical investigations with PF-06804103 have shown substantial control over tumor growth in a variety of tumor models, including breast cancer and HER2-low gastric and esophageal cancers (GC). PF-06804103 showed effectiveness in mice with tumors expressing low to moderate levels of HER2 in HER2-expressing patient-derived xenografts [74].

A phase 1 clinical trial (NCT03284723) involving patients with HER2-positive and HER2-low breast cancer, as well as HER2-positive gastric cancer (GC), was conducted to evaluate PF-06804103. The study aimed to identify dose-limiting toxicity, assess the safety and tolerability of the treatment, and look into PF-06804103’s preliminary anti-tumor activity. PF-06804103 showed promise in improving efficacy in a study that included individuals with HR+ HER2-low and HER2+ breast tumors. The longest period of reaction (DoR) recorded in the research was 18.9 months in a patient receiving PF-06804103 at a dose of 3.0 mg/kg for HER2+ breast cancer. The median DoR among the five verified responders who received 4.0 mg/kg varied from 7.0 to 18.7 months. In both the HR+ HER2-low and HER2+ breast cancer groups in Part 2A of the trial, a greater proportion of patients who received the 4.0 mg/kg dose achieved a higher objective response (OR) than those who received 3.0 mg/kg. In particular, the objective response rate (ORR) in HER2+ breast cancer was higher (47.4%) at the 4.0 mg/kg dose level than in HR+ HER2-low breast cancer (27.3%). This discrepancy could be explained by the fact that HER2+ tumors produce more HER2 receptors, which increases PF-06804103 binding and consequently cancer cell death. These results point to PF-06804103’s possible dose-dependent efficacy in treating HER2+ and HR+ HER2-low breast tumors, which calls for more research in clinical trials [74].

### 5.13. Anetumab-Ravtansine

Anti-HER2 immunoglobulinG1 ADC, PF-06804103, is made up of an anti-HER2 monoclonal antibody (trastuzumab) coupled to the cytotoxic agent Aur0101 at specially designed reactive cysteine sites. Mesothelin is a transmembrane antigen for tumor differentiation that exhibits significant expression in a variety of solid tumors, as determined by immunohistochemistry (IHC). These tumors include mesothelioma (85–90%), pancreatic (80–85%), ovarian (60–65%), non-small-cell lung (57–64%), stomach (50–55%), and breast (25–30%) cancers. Mesothelin may be crucial for tumor implantation and metastasis, although its typical physiologic function is yet to be well understood. An antibody–drug conjugate called anetumab-ravtansine (BAY 94-9343) is made up of a completely human immunoglobulinG1 anti-mesothelin monoclonal antibody conjugated to the tubulin inhibitor DM4, a derivative of maytansine, via a reducible disulfide linker. Antineotab-ravtansine has a drug-to-antibody ratio of 3.2. Anetumab-ravtansine binds to mesothelin on tumor cells, cleaves the disulfide linker, and internalizes the drug to release DM4, followed by DM4 binding to tubulin, which prevents microtubule polymerization, and causes apoptosis subsequent cell cycle arrest. Adjacent dividing cells in the tumor microenvironment are killed due to DM4 being released into it. The main goals of an open-label, non-randomized phase I study with dose escalation and expansion were to ascertain the pharmacokinetics, MTD, safety, and tolerability of anetumab-ravtansine. The analysis of the tumor response, including the best complete response [CR], partial response [PR], and disease control rate (DCR; ideal response of CR, PR, or stable disease [SD]), median progression-free survival (PFS), examination of mesothelin expression, plasma SMRP, and immune response of anetumab-ravtansine were among the secondary objectives. In a phase I trial, anetumab-ravtansine showed early anticancer activity in patients with ovarian cancer and metastatic and refractory mesothelioma. On the other hand, anetumab-ravtansine did not show a greater efficacy than vinorelbine in a randomized phase II research concerning pleural mesothelioma. Tumor mesothelin expression levels correlate with progression-free survival (PFS) and overall survival (OS) by subgroup assessments, which are part of the ongoing analysis from this phase II investigation. These results imply that although anetumab-ravtansine showed early promise, more information is required to completely evaluate its clinical potential in this context. Compared to traditional therapies like vinorelbine, its effectiveness in treating mesothelioma is presently being assessed [75].

### 5.14. OBI-999

OBI-999 is a next-generation antibody–drug conjugate which is a combination of Globo H-targeting antibody (OBI-888) with the cytotoxic agent monomethyl auristatin E (MMAE). In xenograft models of pancreatic, breast, and gastric cancers, as well as a lung cancer patient-derived xenograft model, OBI-999 demonstrated strong, dose-dependent inhibition of tumor growth. OBI-999 selectively binds to Globo H through its humanized monoclonal IgG1 antibody linked to MMAE. In preclinical tests, it was seen that OBI-999 has specificity towards Globo-H marker in tumor cells. Globo H, released by cancer cells, seems to contribute to carcinogenesis by shielding cells from apoptosis, inhibiting immune cell activity, and encouraging angiogenesis. OBI-999 is internalized and transported to lysosomes after attaching to the antigen on cancer cells. There, cathepsin B cleaves the linker, releasing the antimitotic MMAE. OBI-999 is an example of an antibody–drug combination (ADC) that reduces the systemic toxicity of highly effective chemotherapeutic drugs while increasing the anticancer activity of therapeutic antibodies. In a phase 1 clinical trial NCT04084366, the most frequent treatment-emergent adverse events (TEAEs) during the study were neutropenia and anemia, when OBI-999 was introduced as an exclusive therapy, it was well tolerated [76].

### 5.15. BAT-8001

Targeting HER2-expressing cells, BAT8001 is a new ADC made up of a trastuzumab biosimilar product (BAT0606) that binds covalently to batansine (a maytansine derivative) via a strong amide bond, which is not easily cleavable. The non-hydrolyzable linker called 6-maleimidocaproic acid is present in BAT8001 to prevent the release of hazardous payload into the bloodstream. In order to ensure stability, BAT8001’s linker and payload are joined by a strong amide bond. An active metabolite is released when the BAT8001-HER2 complex is endocytosed and broken down in lysosomes following binding to HER2. The toxicity profile of BAT8001, a HER2-targeting antibody–drug conjugate (ADC) with a microtubule inhibitor payload, is comparable to that of T-DM1, mainly due to thrombocytopenia, a known adverse effect of microtubule inhibitors. Using the right drugs and dose reductions across several dose cohorts (3.6 mg/kg, 4.8 mg/kg, and 6.0 mg/kg), grade 3/4 thrombocytopenia episodes were successfully controlled without leading to significant bleeding occurrences. As with T-DM1, hepatic transaminase increase was brief and reversible and was described as a dose-limiting hazard at the highest dose cohort (6.0 mg/kg). Interestingly, this research did not record any cases of pneumonitis, a problem associated with other HER2-targeting ADCs; however, caution in larger patient cohorts is advised. Even though the cohort was extensively pretreated (76% relapsed following HER2 inhibitor therapy, and 66% had ≥3 prior chemotherapeutic regimens), BAT8001 showed potential efficacy in advanced or metastatic HER2-positive breast cancer. Impressive rates of disease management and objective response were seen, with 82.8% and 41.4% of patients reaching these objectives, respectively. Remarkably, a single patient attained complete response (CR) at the maximum tolerated dose (MTD), consistent with T-DM1’s claimed effectiveness. Although the results are strong, further randomized controlled studies are required to confirm the safety and efficacy of BAT8001 due to the study’s diverse and small sample size [77].

### 5.16. Aprutumab-Ixadotin

Aprutumab-ixadotin (BAY 1187982) is a new ADC that consists of an inventive auristatin W derivative and an entirely human anti-FGFR2 monoclonal antibody (BAY 1179470) coupled by lysine side chains to a non-cleavable linker. This new and extremely effective microtubule-disrupting compound, auristatin W derivative, was being employed in an ADC (aprutumab-ixadotin) for the first time. As previously mentioned, lysosomal degradation of the antibody moiety releases the non-cell-permeable payload metabolite, which is unable to pass cell membranes because it contains a charged group. Aprutumab-ixadotin’s low nanomolar efficacy was shown by in vitro research findings, which also revealed a correlation between high FGFR2 expression and internalization as well as cytotoxic effects. In a phase 1 clinical trial (NCT02368951) of aprutumab-ixadotin, FGFR2 tumor expression was the target. Aprutumab-ixadotin was the first ADC to target FGFR2, and the most common treatment-emergent adverse events (TEAEs) among patients treated with aprutumab-ixadotin were thrombocytopenia and increased AST levels, affecting 50% and 60% of the patients, respectively. In this phase I trial, the ADC showed poor tolerability, with an MTD of 0.2 mg/kg, which was lower than the predicted minimum effective dose from preclinical studies. The drug’s safety profile in humans significantly differed from that observed in animal models, with a high rate of nephropathy and proteinuria [78].

### 5.17. Mirvetuximab-Soravtansine (MIRV)

A possible target for ADCs is folate receptor alpha (FRα). Triple-negative breast cancer (TNBC), endometrial cancer (EC), and epithelial ovarian cancer (EOC) fall under this category and exhibit a substantial elevation of FRα. Normal tissues use alternative folate transporters and lack FRα expression. Throughout the course of the malignancy, gynecological tumors with upregulated FRα expression typically remain constant and are linked to a bad prognosis. Patients with poor prognoses and limited therapy options are those with recurrent severely pretreated EOC, EC, or TNBC. An appealing therapeutic approach that may boost the effectiveness of each drug with manageable toxicity is combining a promising targeted therapy, such as MIRV, with a conventional chemotherapeutic agent, such as gemcitabine, with low overlapping toxicity [79]. In TNBC, treatment with mirvetuximab should only be investigated further in the event that a different diagnostic strategy for patient selection is created based on more preclinical data [80].

### 5.18. Lu-177-Trastuzumab

Trastuzumab was conjugated to DOTA, a bifunctional chelator, and its conjugation number and integrity were measured. Radioactive lutetium or Lu-177 was used to optimize the radiolabeling of trastuzumab conjugated with DOTA. This novel combination compound selectively targeted metastases and primary tumors that were HER2 positive. Future research will ascertain whether Lu-177-trastuzumab is a useful therapeutic alternative [81,82].

### 5.19. MM-302-Doxorubicin Conjugate

Liposomal doxorubicin compositions exhibit less cardiotoxicity and fewer adverse effects than free doxorubicin. Targeted antibody conjugates hold significant promise for increasing absorption by cancer cells while reducing systemic toxicity. Doxorubicin could be delivered only to HER2-overexpressing cells, sparing the heart and enabling the use of one of the most potent chemotherapy drugs. MM-302 is a liposomal doxorubicin compound that is PEGylated and targets HER2-overexpressing tumor cells. MM-302also distributes doxorubicin to tumor cells while limiting exposure to healthy cells such as cardiomyocytes. When MM-302 is used alone, in combination with trastuzumab, or combined with cyclophosphamide and trastuzumab, it exhibits a good safety record and encouraging clinical results in advanced HER2-positive breast cancer. MM-302 at 30 mg/m^2^ and trastuzumab at 6 mg/kg was the suggested phase 2 dose chosen every three weeks. The successful completion of the regimen’s initial studies has sparked thoughts about conducting more clinical investigations to support and build upon these positive results [83]. Aiming to investigate whether MM-302, a novel HER2-targeted antibody–liposomal doxorubicin conjugate, combined with trastuzumab could be a safe and effective treatment option for patients who have not taken an antibiotic and have HER2-positive advanced or metastatic breast cancer and who may benefit from systemic therapy after the disease progresses on approved agents, is the purpose of the HERMIONE trial [84].

### 5.20. Praluzatamab-Ravtansine (CX-2009)

A possible target for ADCs is folate receptor alpha (FRα). Triple-negative breast cancer (TNBC), endometrial cancer (EC), and epithelial ovarian cancer A precision-targeted medication called pralizumab-ravtansine (CX-2009) is intended to attach to the transmembrane glycoprotein CD166, which is present in both normal and malignant tissues. Angiogenesis, inflammation, tumor growth and invasiveness, monocyte movement across endothelial tissues, leukocyte intravasation in the central nervous system, T-cell activation, and hematopoiesis are only a few of the physiological processes in which CD166 is essential [85].

CX-2009 is composed of a monoclonal antibody (mAb) linked to *N*2′-deacetyl-*N*2′-(4-mercapto-4-methyl-1-oxopentyl)-maytansine (DM4), a strong inhibitor of microtubules, via a disulfide-cleavable linker. Due to this conjugation, the average drug-to-antibody ratio (DAR) is approximately 3.5, meaning that there are typically 3.5 DM4 molecules for every antibody molecule. By delivering the lethal DM4 payload to CD166-expressing cells only, this focused strategy may increase therapeutic efficacy while reducing off-target consequences. The goal of a phase I/II trial (CTMX-M-2009-001; NCT03149549) was to determine the recommended phase II dose (RP2D) by assessing the tolerability, activity, and pharmacokinetics of CX-2009 [85].

The study offers indirect proof of tumor-associated protease activity and the Probody platform’s value while demonstrating an appropriate safety profile for CX-2009 that is commensurate with previous DM4 ADCs. Probody stands for proteolytically activated antibody. These antibodies are engineered to stay inert until these are activated locally in the diseased tissues. Principally, any therapeutic antibody can be switched to its Probody form. Probody was successful in dosing escalating CX-2009 to levels that were physiologically active within a tolerable therapeutic range. Although CD166 expression has been linked to the grade, stage, and invasive potential of various tumors, including breast cancer, it has not been shown to be a reliable indicator of how well a patient would respond to this therapy. By enrolling patients whose tumor types are projected to have high levels of CD166 using an experimental IHC assay, this trial is the first to assess the predictive ability of CD166 to support treatment with CX-2009. A phase II trial (NCT04596150) is being conducted to evaluate the effectiveness of CX-2009 as a single treatment in patients with TNBC and HER+/HER2-breast cancer. Additionally, the trial aims to evaluate the combination of CX-2009 with CX-072, a Probody therapy that targets PD-L1, in patients with TNBC [85].

### 5.21. Trastuzumab (LCB ADC 1 and 2)

MMAF is an analog of dolastatin that inhibits cell division by disrupting tubulin polymerization, hence exerting known anti-tubulin and antineoplastic properties. The LCB-ADCs had MMAF as their poisonous ingredient because it was intended to be significantly more effective when incorporated into cells via an antibody. The C-terminal carboxyl group of the compound contains a binding site where a dipeptide linker can be attached to enzymes that can catalyze drug release. This connection can be used to modify the drug’s potency, effectiveness, and tolerance. Unlike T-DM1, LCB-ADCs are made to achieve a consistent DAR of 2 or 4, meaning that they are stable in plasma and can prolong the drug’s half-life significantly. They also have a cleavable linker that is intended to facilitate higher efficiency through various mechanisms, including the bystander effect. The anticancer efficaciousness of LCB-ADCs was dramatically enhanced in patients with both overexpressed and low-expressed HER2. The bystander effects of MMAF, which is membrane permeable and can therefore enter and kill neighboring cells in contrast to other agents used in ADCs like monomethyl auristatin E (MMAE), which remains stuck in the cell, may also contribute to the more substantial anticancer effect of LCB-ADCs [86].

### 5.22. Depatuxizumab-Mafodotin (Depatux-m)

A possible target for ADCs is folate receptor alpha (FRα). Triple-negative breast cancer (TNBC), endometrial cancer (EC), and epithelial ovarian cancer changes in the epidermal growth factor receptor (EGFR) have been linked to several malignancies. Although they have improved efficacy, the current EGFR-directed treatments have certain negative (side) effects. The highly tumor-specific antibody–drug combination depatuxizumab-mafodotin (depatux-m) targets EGFR via a monoclonal antibody coupled to a cytotoxin [87]. Depatuxizumab (depatux) (previously ABT-806) is a humanized, recombinant immunoglobulinG1κ (IgG1κ) monoclonal antibody that targets a distinct conformation of human EGFR that is exposed because of either a high level of gene amplification, EGFR variant III (EGFRvIII) deletion of exons 2 through 7, or EGFR overexpression (increased receptor density) [87]. The study of M13-379 (https://clinicaltrials.gov/search?term=NCT01741727, accessed on 13 October 2024) aimed to assess the safety, PKs, and effectiveness of depatux-m in patients with advanced solid tumors. The study was planned as an open-label phase 1 and 2 trial. Patients with advanced solid tumors in the phase 1 research showed that depatux-m was typically well tolerated. Both the EGFR-targeting monoclonal antibody (depatux) and the ADC that uses it (depatux-m) showed negligible binding in healthy tissues and did not cause additional toxicities. Mafodotin, a strong microtubule inhibitor linked to ocular adverse effects, is the payload of DepatuxTM. Ocular adverse events (AEs) have also been documented in other ADCs trials using mafodotin with various targeting antibodies [87].

### 5.23. Losatuxizumab-Vedotin

Targeting the epidermal growth factor receptor, losatuxizumab-vedotin (formerly known as ABBV-221) is a second-generation antibody–drug combination. Losatuxizumab-vedotin is an ADC targeting EGFR found on cancer cells. Losatuxizumab-vedotin comprises an affinity-matured antibody that binds specifically to active EGFR, a cathepsin-cleavable valine-citrulline (vc) linker, and the potent microtubule inhibitor MMAE. Upon binding to EGFR, the ADC is internalized where the vc linker is cleaved, releasing MMAE. This disrupts cell division, leading to cancer cell death. Unlike depatux-m, losatuxizumab-vedotin uses MMAE, which is known for minimal ocular toxicity in other ADCs like brentuximab-vedotin. Losatuxizumab-vedotin has a higher affinity for both wild-type and mutant EGFR, potentially targeting a broader range of tumors effectively. Patients treated with losatuxizumab-vetotin did not experience any notable ocular toxicity, in contrast to depatux-m. In this trial, infusion responses were found in 48.9% of patients; these reactions did not seem to be connected to dosage; 21 patients (46.7%) had an infusion reaction after receiving their first dose. It is interesting to note that 95% of individuals who had an infusion reaction were able to withstand additional infusions of losatuxizumab-vedotin without experiencing any more reactions. Four patients (8.9%) had an infusion reaction of grade 3 or higher despite the fact that the majority of infusion reactions were mild to moderate. The study was registered on ClinicalTrials.gov (Trial registration ID: NCT02365662) [88].

### 5.24. Rolinsatamab-ABBV-176

The humanized antibody h16f (PR-1594804) is combined with a highly effective and cytotoxic crosslinking pyrrolobenzodiazepine dimer (PBD; SGD-1882) to form ABBV-176, an antibody–drug combination that targets the prolactin receptor (PRLR), which is overexpressed in several solid tumor types. In patients with advanced solid tumors anticipated to show increased PRLR levels, the safety, pharmacokinetics, and preliminary activity of ABBV-176 were assessed in the stage 1 dose-escalation study (NCT03145909). Phase 1 dose-escalation investigation revealed severe toxicity related to ABBV-176. Even while cytopenias frequently had a dose limit, effusions and oedema (edema) were also frequent and showed signs of cumulative toxicity due to their late start. Although the information was obtained from a limited number of patients with varying tumor PRLR expression, no responses were seen. This trial was stopped after 19 volunteers had received their doses [89].

### 5.25. BR96-Doxorubicin Immunoconjugate

A chimeric human/mouse monoclonal antibody called BMS-182248-1 (BR96-doxorubicin immunoconjugate) is covalently bonded to approximately eight doxorubicin molecules [90]. When BR96-dox binds to the Lewis-Y (LeY) antigen, the complex quickly internalizes, and doxorubicin is released intracellularly through acid hydrolysis in the lysosomes’ and endosomes’ acidic environments. Doxorubicin then localizes to the nucleus, where it inhibits cell division and causes DNA intercalation. The molar ratio of the drug to antibody in the BR96-dox is approximately 8:1, meaning that each antibody molecule binds to eight doxorubicin molecules [91]. The LeY antigen, which is limited in expression on normal tissues but expressed on 75% of all breast tumors, is the target of the antibody. In a study population with demonstrated sensitivity to single-agent doxorubicin, a phase II design was selected to assess the activity of the BR96-doxorubicin combination in metastatic breast cancer. In patients with detectable metastatic breast cancer and immunohistochemistry evidence of LeY expression on the tumor, doxorubicin 60 mg/m^2^ every three weeks or BR96-doxorubicin conjugate 700 mg/m^2^ IV was administered. There were twenty-three assessable patients with a median of one prior chemotherapy course. In 14 patients receiving the BR96-doxorubicin combination, there was one partial response (7%); while in 9 assessable patients receiving doxorubicin, there was 1 complete response and 3 partial responses (44%). A hypersensitive reaction that was clinically severe did not occur in any patient. The two therapy groups showed considerable differences in toxicities. The hematologic toxicities in the BR96-doxorubicin conjugate group were modest, but the gastrointestinal toxicities were prominent and included marked increases in serum lipase and amylase, nausea, and vomiting associated with gastritis. While BR96-doxorubicin immunoconjugate targets a specific antigen, it binds to normal tissues with the same antigen, leading to gastrointestinal toxicities and compromising its ability to effectively reach and treat metastatic breast cancer tumors [90].

### 5.26. DLYE5953A

The glycophosphatidylinositol-anchored, interferon-inducible surface membrane protein known as LY6E is encoded by the lymphocyte antigen 6 complex locus E gene (LY6E). This protein has been discovered as a potential target for ADCs. While most normal tissues have low levels of LY6E expression, common epithelial malignancies, such as breast cancer (64%), pancreatic cancer (63%), non-small-cell lung cancer (56%), and ovarian cancer (53%), commonly have high levels of LY6E expression (IHC 2/3+). DLYE5953A is an ADC that consists of a strong antimitotic drug (monomethyl auristatin E, or MMAE) and a humanized anti-LY6E monoclonal antibody (IgG1: MLYE4489A) connected by a protease-labile linker (maleimidocaproyl-valine-citrulline *p*-aminobenzyloxycarbonyl). DLYE5953A is internalized and transported to lysosomes, where MMAE or MMAE-containing catabolites cause cell death after binding to LY6E on the cell surface. ADCs can, therefore, minimize drug exposure to normal tissue while increasing drug exposure, especially to cancer cells that express the target relatively highly. DLYE5953A demonstrated strong and specific suppression of cell growth in LY6E-expressing cancer cell lines and mouse xenograft models throughout preclinical investigations. Phase I study DLYE5953A, an anti-LY6E ADC, was found to have an acceptable safety profile when given by IV infusion Q3W to patients with solid tumors, including MBC and NSCLC. The study was registered on ClinicalTrials.gov (NCT02092792) and evaluated safety, tolerability, RP2D, PK, anti-drug antibody (ADA) formation, preliminary anti-tumor activity, and potential biomarkers [92].

For DLYE5953A, no dose-limiting events (DLTs) were found in dosage-escalation cohorts up to the maximum given dose of 2.4 mg/kg Q3W. The RP2D for DLYE5953A was found to be 2.4 mg/kg Q3W, even though a protocol-defined MTD was not met. This value has been chosen for several other ADCs delivering MMAE with comparable drug-to-antibody ratios. DLYE5953A’s safety profile matched with earlier findings for ADCs that used MMAE as the cytotoxic agent. Nonetheless, there are some noteworthy variations in the safety profile of DLY5953A, such as alopecia rates and IRRs. Of all patients, 54% had alopecia, which is almost 2 to 3 fold more than other vc-ADCs containing MMAE [92].

### 5.27. Disitamab-Vedotin

Disitamab-vedotin, also known as RC48-ADC, is a type of antibody–drug conjugate which targets HER2+ BC. Disitamab-vedotin consists of a cleavable linker that is attached to a synthetic cytotoxic agent monomethyl auristatin E or MMAE, which works by disrupting the microtubules of cancer cells [93]. The drug-to-antibody ratio (DAR) for disitamab-vedotin is 4. Disitamab-vedotin’s mechanism of action is binding to the HER2 protein on the membrane of a cancer cell and then leads to endocytosis, where the payload is released into the lysosomes killing the cancer cells [94]. A phase 1 first-in-human study, C001 CANCER NCT02881138, and a phase 1b open-label dose-escalation trial, C003 cancer NCT03052634, in patients with HER2-positive metastatic breast cancer showed that patients in both the studies had locally advanced or metastatic HER2-positive breast cancer with IHC 3 or IHC 2-positive with FISH amplification. The C001 cancer trial was an open-label parallel trial using a 33 dose-escalation design performed to find out the MTD among the doses of 0.5, 1.0, 1.5, 2.0 and 2.5 mg/kg. The C003 open-label CANCER trial was conducted parallelly and was performed to identify the accurate phase 2 dose through trial of three dosage levels, 1.5, 2.0 and 2.5 mg/kg, once every two weeks. Acceptable tolerance and promising efficacy of RC48-ADC was seen in patients with HER2-positive MBC when administered at the 2.0 mg/kg once every two weeks dose level and was identified as the most beneficial because of the balance between the benefit and risk among the other dosage levels. A phase 2 study NCT03500380 is being conducted to compare the efficacy of RC48-ADC administered 2.0 mg/kg once every two weeks when compared to Capecitabine plus Lapatinib in the treatment of HER2-positive MBC [95].

### 5.28. Ladiratuzumab-Vedotin

LIV-1, or SLC39A6, is a zinc transporter protein that is expressed in BC, and it was first identified as an estrogen-inducible gene in cell lines established from breast cancer. LIV-1 promotes the epithelial to mesenchymal transition, which is a pivotal step in the progression of malignancy towards metastasis. By using IHC (immunohistochemistry) studies, it has been reported that LIV-1 expression happens in ER+/HER2−, HER2+, and TNBC metastatic breast cancer. The expression of LIV-1 is confined to four hormonally regulated organs including breast, prostate, uterus, in healthy human tissues. The expression of LIV-1 is widespread in metastatic breast cancer but is poorly expressed in non-hormonally regulated organs, making it a target for ADCs. A humanized IgG1-based ADC targeting the LIV-1 protein is ladirituzumab-vedotin, also known as SGN-LIV1A. The MMAE cytotoxic payload in ladirituzumab-vedotin is delivered, and favors cytotoxic tumor cell death for enhanced anticancer activity. The drug and the antibody are linked via a protease cleavable linker [96]. In a phase Ib/II study in patients with mTNBC (NCT03310957), ladiratuzumab-vedotin in combination with pembrolizumab, was evaluated for safety, tolerability, activity, and RP2D. In total, 51 patients were enrolled on this trial, 44 of whom received SGN-LIV1A at the RP2D of 2.5 mg/kg. The most frequent adverse events at grade 3 or worse included tiredness, hypokalemia, diarrhea, neutropenia, and maculopapular rash, 8% each. Of 26 patients treated whose response could be assessed, the ORR was 54%. In summary, the study validated the potential effectiveness of this treatment regimen for patients with mTNBC and its tolerability [94].

### 5.29. AVID100

AVID100 is a novel carefully crafted and innovative antibody–drug conjugate that targets the epidermal growth factor receptor (EGFR) exclusively. EGFR is highly expressed in many different types of cancer, and becomes an easy target for ADCs. However, on-target off-tumor toxicity is a problem because normal skin cells also contain EGFR. Anti-EGFR-DM1 conjugation AVID100 is very effective against cancer cells compared to unconjugated antibodies. However, it does not show any enhanced toxicity towards normal cells. It was observed that the AVID100 antibody moiety, known as MAB100, competes with EGF for binding to the EGFR and has a comparatively high affinity for the receptor (approximately 2 nM). Furthermore, MAB100 also blocks EGFR’s downstream signaling as seen by a higher rate of apoptosis in AVID100-treated tumor cells compared to MAB100-treated tumor cells. MAB100 not only displayed full antagonist efficacy but also successfully delivered the microtubule inhibitor DM1 to tumor cells. When it came to tumor cell lines generated from lung, head and neck, and breast malignancies, AVID100 was very efficient [97]. AVID100’s activity was examined in a number of mouse xenograft trials, including FaDu SSCHN, H292 NSCLC, and MDA-MB-468 human breast cancer models. Even when given as a single dose, AVID100 therapy dramatically slowed tumor growth and produced tumor regressions in some of the mice. Studies on the toxicology of cynomolgus monkeys showed that AVID100 was well tolerated at doses of up to 10 mg/kg up to four times a week. After AVID100 underwent a phase 1 clinical investigation, the NCT03094169 clinical trial is now conducting the AVID100 phase 2 trial [97].

### 5.30. ARX-788

ARX-788 is a newly developed ADC which comprises an exquisite anti-HER2 mAb targeting HER2+ breast cancer. ARX-788 carries a potent cytotoxic payload AS269, which is mainly a tubulin inhibitor that forms a strong bond with the antibody, thereby limiting the growth of the tumor upon delivery without damaging healthy tissues. The mAb is linked to AS269 via a para-acetylphenylalanine (pAF). In preclinical studies, ARX-788 demonstrated a positive response in HER2-low and T-DM1 resistant tumors [19]. Analyzing phase 1, both the ACE-Breast-01 trial and the ACE-Pan-tumor-01 trial have shown 100% DCR, while the ORR for 1.5 mg/kg was observed as 74% (14/19) and 67% (2/3) in both the trials, respectively. ARX-788 also showed lower systemic cytotoxicity, which was mostly because of ARX-788 being very stable and less serum exposure to the pAF-AS269 moiety [98]. An open-label phase II clinical trial currently being conducted is on patients with MBC heavily pretreated with T-DXd. Another trial is also continuing the I-SPY2 trial (NCT01042379), which involves treatment with conjugation therapy of ARX-788, with cemiplimab being a PD1 inhibitor in early-stage breast cancer [19].

### 5.31. XMT-1522

For patients with HER2+ breast cancer who frequently show resistance to trastuzumab-emtansine, XMT-1522 can be used as an alternative treatment. XMT-1522 consists of 10–15 molecules of the payload AF-HPA, which is an auristatin derivative coupled to a new anti-HER2 monoclonal antibody using the Dolaflexin ADC platform. AF-HPA has a two-step intratumor metabolism, which is aimed at optimizing therapeutic index of the ADC treatment. AF-HPA does not compete with trastuzumab for HER2 binding; instead, it binds to an epitope different from the trastuzumab-binding site on domain IV of HER2. Each antibody in XMT-1522 contains an average of 12 auristatin F-hydroxypropylamide (AF-HPA) moieties, and these 12 moieties are connected to HT-19 by a cysteine linkage made of a hydrophilic polymer that breaks down naturally, which allows for high AF-HPA loading. The preclinical trial exhibited xenograft tumor models like JIMT-1 and RN-87, progressed while receiving T-DM1 treatment. Alternatively, every tumor responded to XMT-1522, and all but one tumor were wiped out while receiving XMT-1522 treatment. When T-DM1 was used to advance RN-87 and JIMT-1 xenografts, XMT-1522 demonstrated a potent anticancer impact. Hence, the conclusion was drawn that XMT-1522 worked well in T-DM1-resistant xenograft models, gastric cancer cell lines, and HER2-positive breast cancer cell lines [99]. The phase 1 trial of XMT-1522 is underway in clinical trial NCT02952729.

### 5.32. ALT-P7

ALT-P7 is a therapeutic antibody–drug conjugate that is the variant of trastuzumab, wherein the cysteine-containing antibody is bound to two molecules of MMAE, which targets the tubulin at a specific site. In a phase 1 3+3 dose-escalation study, NCT03281824, patients with HER2-positive breast cancer who had been treated very heavily with a minimum of two rounds of anti-HER2 therapy were considered for this trial. A total of 27 patients were administered a single dose of IV injection at doses ranging from 0.3 mg/kg to 4.8 mg/kg every three weeks. Neutropenia, myalgia, fatigue, alopecia, and pruritus were the common toxic effects. Three of them had dose-limiting toxicities of 4.8 mg/kg. The disease control rate after six weeks of ALT-P7 treatment was 77.3%, while progression-free survival was 6.2 months, applying a dosage between 2.4 mg/kg and 4.8 mg/kg on average. ALT-P7 possesses high tolerability potential along with the ability to control the disease [100].

### 5.33. Zanidatamab-Zovodotin

Zanidatamab-zovodotin, also known as ZW49, is a bispecific antibody–drug conjugate (ADC) designed to target HER2-positive cancers by recognizing two distinct, non-overlapping sites on the HER2 protein. This ADC consists of a bispecific antibody attached to an auristatin toxin, a compound that disrupts cancer cell growth. An ongoing clinical trial (NCT03821233) aims to evaluate the drug’s effectiveness and safety profile in its first-in-human phase I study. The main objectives of this trial are to assess the anticancer activity of zanidatamab-zovodotin when used alone, determine the maximum tolerated dose (MTD), and document its safety and tolerability in patients with HER2-positive solid tumors. The drug has shown promise, with phase 1 results revealing an acceptable safety profile when administered at a dose of 2.5 mg/kg every three weeks (Q3W) via intravenous infusion. Most of the side effects were mild and manageable, with the most frequent ones being grade 1 or 2 diarrhea, hair loss (alopecia), and eye inflammation (keratitis). Importantly, no deaths or severe toxicities were reported during the dose-escalation phase, and there were no dose-limiting toxicities. In terms of effectiveness, zanidatamab-zovodotin showed an objective response rate (ORR) of 13%, with a partial response (PR) rate of 13%, indicating promising anticancer activity [101].

### 5.34. Camidanlumab-Tesirine (ADCT-301)

The tumor microenvironment becomes immunosuppressive because of the presence of regulatory T lymphocytes or (Tregs). They also constitute a first line of resistance against tumor elimination by immunotherapies and are involved in the development and progression of tumors with high Treg infiltration that shows an imbalance between Treg and Teffs. Although they have not been successful in all cases, many strategies have been attempted to decrease or prevent Tregs. ADCT-301 or camidanlumab-tesirine is an ADC that has a DAR of 2.3 consisting of the HuMax-TAC, a human IgG1 mAb targeting human CD25, attached to a potent pyrrolobenzodiazepine (PBD) dimer payload SG3199 through a cathepsin-cleavable valine-alanine peptide linker. PBD dimers offer non-distortive interstrand crosslinks in the minor groove of DNA, which are resistant to DNA repair and allow the DNA interstrand crosslinks to survive, giving them a particular advantage over other warheads. This ADC targets CD25-positive tumor cells with a high infiltration of Tregs and destroys it along with the help of checkpoint inhibitors [102].

### 5.35. FS-1502

FS-1502 is a monoclonal antibody conjugated to MMAF (monomethyl auristatin F) by a β-glucuronide linker targeting HER2+ breast cancer. MMAF acts by inhibiting the polymerization of tubulin. The β-glucuronide linker being cleavable enables a payload release that is mediated and does not impact normal cells and tissues as it acts primarily on targeted tumor cells only. In xenograft models and in vitro studies, FS-1502 had more target-specific activity in comparison to T-DM1 and had reasonably high anti-tumor activity in breast tumor models. In addition to this, FS-1502 performed very well in limiting the growth of HER2-low tumors that were T-DM1 resistant in xenograft models.

In a phase 1 clinical trial NCT03944499, an open-label study to assess the safety, tolerability, and anti-tumor response of FS-1502, it was observed that there were drug-related TEAEs such as an increase in levels of AST and ALT along with hypokalemia and low cardiac toxicity in 146 patients out of 150 patients. The dosage level of 1.0 mg/kg showed a ORR (objective response rate) of 53.7% and remarkable anti-tumor responses were observed in HER2-low BC with an ORR of 26.1% [103].

## 6. Demand for Next-Generation ADC

ADCs have made significant strides in recent years. Next-generation ADCs have achieved notable advancements in optimizing antibodies, innovating linkers, and selecting payloads. The development of humanized and engineered monoclonal antibodies has improved tumor targeting, while cleavable linkers enable more precise release of the drug payload. Additionally, highly potent, membrane-permeable cytotoxic agents have enhanced bystander effects and treatment effectiveness. These improvements have widened the applications of ADCs, increased therapeutic efficacy, and reduced adverse event rates. At present, over 10 next-generation ADCs are undergoing clinical trials, aiming to become viable second-line treatments for advanced HER2-positive breast cancer [65]. Bispecific ADCs (BsADCs) are a unique type of ADC engineered by joining the advantages of ADC along with bispecific antibodies, which can deal with the challenges faced by normal ADC, such as penetrating the tumor, resistance to drugs, and off-target effects. BsADCs can enter the tumor through two types of endocytosis processes: clathrin mediated and clathrin non-mediated. The conjugation of FcRn to Fc is the main step towards stabilizing the ADC in systemic circulation by avoiding premature degradation. The characterization of BsADCs is performed based on their binding pattern. They can bind to two different antigens or two distinct epitopes on a common antigen. This binding pattern influences the mechanism of BsADCs in the body, improves specificity, inhibits the signaling cascade, and avoids drug resistance. Tumor antigens that are expressed at low levels can be detected by the use of TCRm antibodies. This is a type of novel BsADC, where TCRm antibodies are linked to monomethyl auristatin E with a DAR of 1.2. Many BsADCs, such as ZW49 and MEDI 4276, are under phase 1 or 2 clinical trials [106]. Combination treatment of ADC and immunologically active drugs can delay drug resistance. These immunotherapies include anti-estrogen therapy and the use of CDK4 or -6 inhibitors. The next generation of antibody–drug conjugates (ADCs) is under development and introduces various innovative approaches to enhance cancer treatment. These include bispecific ADCs, which target two different antigens on cancer cells for improved specificity, and pro-body drug conjugates, which remain inactive until activated by proteolytic enzymes in the tumor microenvironment, minimizing off-target effects. Additionally, radionucleotide–drug conjugates combine targeted radiation with cytotoxic drugs to deliver a dual attack on tumors, while immunostimulatory ADCs not only kill cancer cells but also activate the immune system for a more robust anti-tumor response. Lastly, small-molecule drug conjugates use smaller compounds instead of large antibodies, allowing better tumor penetration and reducing immune system recognition. These advancements aim to improve treatment precision, effectiveness, and patient outcomes [107]. In conclusion, introducing next-generation ADCs such as BsADCs has transformed breast cancer treatment and research. Ongoing studies and regulatory approvals highlight the promising role that ADCs will continue to play in combating the disease. As further advancements unfold, ADCs are expected to deliver more targeted and effective treatments, improving patient survival rates and quality of life [65].

## 7. Conclusions

Combining powerful cytotoxic medicines with monoclonal antibodies, antibody–drug conjugates (ADCs) represent a promising class of targeted cancer therapeutics. By explicitly targeting antigenic markers on cancer cells, ADCs have shown considerable efficiency in treating various cancers. This allows for delivering strong cytotoxic medicines to tumor areas while protecting healthy tissues. Safety improvements, especially regarding linker technology, have reduced off-target effects and improved circulation stability, improving clinical profiles. Several ADCs have received regulatory approval, demonstrating the therapeutic promise of these drugs in actual clinical settings. However, issues still need to be resolved to increase the efficacy of ADCs and expand their potential applications in oncology and other disease areas. Although ADCs are considered a promising therapy to treat breast cancers [19,108,109], some limitations exist, such as loss of tumor-associated antigens, high cost and low supply of payloads, antigen heterogeneity, resistance mechanisms, unavailability of chimeric antibodies, and manufacturing complexities. For example, (i) malignant cells may generate drug resistance [110] against ADCs. Drug resistance may arise if the target changes/modifies itself to avoid interaction with the ADC, or malignant cells may find a new route to proliferate, which is independent of the biomolecular target; (ii) some small-molecule drugs are challenging to synthesize due to their 3D-stereochemical features, resulting in high costs and low supply of these drugs in the market; (iii) humanized monoclonal antibodies for all type of cancers are not available and consequently targeted therapy via ADC is unavailable for such types of cancers; (iv) toxicity towards healthy cells may result in various side effects including hepatotoxicity, cardiotoxicity, nephrotoxicity, and hemotoxicity, which result in liver diseases, diarrhea, high blood pressure, mouth sores, nail changes, and hair loss [15]. Some of these issues are being addressed through continuous research endeavors, while the rest require more in-depth research. This review focuses on the target specificity, chemical names, structures, stereochemical features, and nature of the drugs used to form the ADCs, various dose-related observations, their specificity and overall efficiency, and a detailed discussion of the related toxicity and survival. The key takeaway is that ADCs have demonstrated efficacy precisely because they minimize damage to healthy tissues while specifically targeting cancer antigens. Recent developments in linker technology have improved safety profiles. Clinical approval for a number of ADCs has confirmed their potential as therapeutics. Still, there are issues, including production complexity and antigen variability. As the landscape of breast cancer treatment continues to evolve, the future directions of ADC development hold significant promise. Future research may be consider the following: (i) novel payload development exploring computer-assisted strategies that lead to improved protein-ligand interactions and molecular dynamics that can lead to drug molecules with higher potency with reduced side effects. (ii) The development of ADCs against BC has primarily focused on established targets, such as HER2 and trophoblast antigen 2 (Trop-2), but potential targets like CD44, folate receptor alpha, and other tumor-associated antigens that are overexpressed in specific breast cancer populations should also be focused upon. (iii) Advances in proteomics and genomics enable the discovery of novel biomarkers, which can be used for ADC development. (iv) Advancements in linker technology can also be beneficial in developing more potent ADCs. At present, linkers are classified as cleavable and non-cleavable, and each linker possesses certain advantages and limitations. Advancements in linker technology can explore the potential of biorthogonal chemistry in enhancing the stability of ADCs outside the cells while precisely releasing the cytotoxic drug (payload) in the tumor microenvironment only upon internalization by target cells—and to do this, stimuli-responsive linkers can be a better option. (v) There are various possibilities of combination therapy that involve synergistic combination of ADCs with other forms of therapy such as another drug or immune checkpoint inhibitors. (vi) Prioritization of personalized medicine approaches such as biomarker-driven approaches, genomic profiling, and liquid biopsies. Future initiatives might also include improving manufacturing procedures for broader clinical use, investigating combination therapy, finding predictive biomarkers, extending applications beyond oncology, and refining ADC components for increased efficacy and safety.

## Figures and Tables

**Table 1:** A bird’s eye view of various ADCs targeted to breast cancer.

Entry	Antibody	Drug (Payload) (Figure 2)	Target	Clinical Trial Phase	Reference(s)
1	Trastuzumab (herceptin)	Emtansine (mertansine)	HER2-positive breast cancer	Phase 3 (Approved)	[20,21,22,23,24,25,26,27,28]
2	Trastuzumab	Deruxtecan (DX-8951)	HER2-low breast cancer	Phase 3 (Approved)	[29,30,31,32,33,34,35,36,37,38,39,40,41,42,43,44,45,46]
3	Sacituzumab	Govitecan (SN-38)	Human trophoblast cell surface antigen-2 (Trop-2)	Phase 3 (Approved)	[47,48,49,50,51,52,53,54,55,56,57,58,59,60]
4	Patritumab	Deruxtecan (DX-8951)	Human epidermal growth factor receptor 3 (HER3)-positive breast cancer	Phase 1/2	[61]
5	Thiomab	Pyrrolo[2,1-*c*][1,4] benzodiazepine (PBD) dimer	HER2-positive and HER2-low breast cancer	Phase 1	[62]
6	Datopotamab	Deruxtecan (DX-8951)	Metastatic triple-negative breast cancer (TNBC) and HR+/HER2- metastatic breast cancer	Phase 3	[63]
7	Dolasynthen B7-H4 Directed ADC	Auristatin F-Hydroxypropylamide	Immune-suppressive protein B7-H4 (VTCN1) is overexpressed in endometrial, ovarian, and breast cancers	Phase 1	[64,65]
8	Trastuzumab	TLR7 agonist	TLR7 agonist conjugated to anti-HER2 antibody targeted to HER2+ breast cancer	Phase 1	[66]
9	hL49	Monomethyl auristatin E (MMAE or vedotin)	Melanotransferin CD228 (cell surface protein) in TNBC	Phase 1	[67]
10	Trastuzumab	Dduocarmazine (duocarmycin)	HER 2 + breast cancer	Phase 3	[68]
11	Farletuzumab	Eribulin mesylate	Folate receptor α (FRα)-expressing tumor cells	Phase 1	[69]
12	MEDI4276 (derivative of trastuzumab)	AZ13599185 (tubulysin analog)	Two non-overlapping epitopes in subdomains 2 and 4 of the HER2 ecto-domain	Phase 1	[70]
13	Glembatumumab	Monomethyl auristatin E (MMAE or vedotin)	Glycoprotein NMB expression	Phase 2	[71,72]
14	Cofetuzumab	Auristatin-0101 (Aur0101)	Notch3 expression	Phase 1	[73]
15	Trastuzumab	Auristatin-0101 (Aur0101)	HER2+ breast cancer	Phase 1	[74]
16	Anetumab	Ravtansine or soravtansine (DM4)	Mesothelin-expressing solid tumors	Phase 1	[75]
17	Anti-Globo H antibody	Monomethyl auristatin E (MMAE or vedotin)	Globo H, a glycosphingolipid, overexpressed in cancers of epithelial origin HER2 positive	Phase 1	[76]
18	Humanized anti-HER2 antibody	Mertansine (DM1)	Breast cancer	Phase 1	[77]
19	Aprutumab	Ixadotin	Fibroblast growth factor receptor type 2 (FGFR2)	Phase 1	[78]
20	Mirvetuximab	Ravtansine or soravtansine (DM4)	Folate receptor alpha (FRα) in TNBC	Phase 2	[79,80]
21	Trastuzumab	Mertansine (DM1)	HER2+ breast cancer	Phase 2	[81,82]
22	scFv anti-HER2	Doxorubicin (adriyamycin)	HER2+ breast cancer	Phase 1	[83,84]
23	Praluzatamab	Ravtansine or soravtansine (DM4)	CD166 transmembrane type-1 glycoprotein	Phase 1/2	[85]
24	Trastuzumab	Monomethyl auristatin F (MMAF)	HER2+ breast cancer	Phase 1	[86]
25	Depatuxizumab	Mafodotin (MC-MMAF)	Epidermal growth factor receptor (EGFR)	Phase 1/2	[87]
26	Losatuxizumab	Monomethyl auristatin E (MMAE or vedotin)	EGFR	Phase 1	[88]
27	Rolinsatamab	Talirine (a crosslinked pyrrolobenzodiazepine dimer (SGD-1882))	Prolactin receptor in solid tumor	Phase 1	[89]
28	BR96 (a chimeric human/mouse monoclonal antibody)	Doxorubicin (adriyamycin)	Lewis-Y antigen, which is expressed in 75% of all types of breast cancers	Phase 2	[90,91]
29	Anti-LY6E	Monomethyl auristatin E (MMAE or vedotin)	LY6E cell surface antigen in HER2-negative metastatic breast cancer	Phase 1	[92]
30	Disitamab	Monomethyl auristatin E (MMAE or vedotin) RC48-ADC	HER2+ breast cancer	Phase 3	[93,94,95]
31	Ladiratuzumab	Monomethyl auristatin E (MMAE or vedotin)	LIV-1 in ER+ breast cancer and TNBC	Phase 1b/2	[94,96]
32	AVID100 (MAB100)	Mertansine (DM1)	Epidermal growth factor receptor (EGFR)	Phase 2	[97]
33	ARX-788 mAb	Amberstatin-269 (AS269 or PEG4-aminooxy-MMAF)	HER2+ breast cancer	Phase 2	[19,98]
34	XMT-1522 (HT-19)	AF-HPA auristatin derivative	HER2+ breast cancer	Phase 1	[99]
35	Trastuzumab biobetter HM2 ALT-P7	Monomethyl auristatin E (MMAE)	HER2+ BC and EGFR	Phase 1	[100]
36	Zanidatamab-Zovodotin (ZW49)	Auristatin payload (ZD02044)	ErbB/HER Family of Receptor Tyrosine Kinases	Phase 1	[101]
37	Camidanlumab-Tesirine (ADC T-301) CD25 targeting mAb	Pyrrolobenzodiazepine (PBD) dimer	CD25 (IL2RA—interleukin 2 receptor alpha subunit, IL-2RA, TAC, p55)	Phase 2	[102]
38	Trastuzumab FS-1502	Monomethyl zuristatin F	HER2+ breast cancer	Phase 1	[103]

**Table 2:** Various clinical trials conducted with T-DM1 and T-DXd [105].

ADC	Antibody	Payload	Representative Trials	Phase	Approval Status
T-DM1	Trastuzumab	DM1	EMILIA (NCT00829166)	III	Yes
			KATHERINE (NCT01772472)	III	
			KATE3 (NCT04740918)	III	
T-DXd	Trastuzumab	DXd	DESTINY-Breast-03 (NCT03529110)	III	Yes
			DESTINY-Breast-09 (NCT04784715)	III	
			DESTINY-Breast-04 (NCT03734029)	III	
			DESTINY-Breast-07 (NCT04538742)	I/II	
			DESTINY-Breast-08 (NCT04556773)	Ib	
