# Correction: Mukherjee, A.; Bandyopadhyay, D. Targeted Therapy in Breast Cancer: Advantages and Advancements of Antibody–Drug Conjugates, a Type of Chemo-Biologic Hybrid Drugs. *Cancers* 2024, *16*, 3517

**DOI:** 10.3390/cancers17101633

**Published:** 2025-05-12

**Authors:** Attrayo Mukherjee, Debasish Bandyopadhyay

**Affiliations:** 1 School of Biotechnology, Kalinga Institute of Industrial Technology (KIIT), Patia, Bhubaneswar 751024, Odisha, India; attrayomukh@gmail.com; 2 School of Integrative Biological and Chemical Sciences (SIBCS), University of Texas Rio Grande Valley, Edinburg, TX 78539, USA; 3 School of Earth, Environmental, and Marine Sciences (SEEMS), University of Texas Rio Grande Valley, Edinburg, TX 78539, USA

In the original publication [1], there are three typographical errors (naming errors). Two are in Table 1, and one is in the text (Section 5.4.) as published. The corrected names are written below.

The authors state that the scientific conclusions are unaffected. This correction was approved by the Academic Editor. The original publication has also been updated.


**Position in the Publication**

**As Appeared in the Publication**

**To Be Read as**
Table 1. Entry 7, Column 2TrastuzumabDolasynthen B7-H4 Directed ADCTable 1. Entry 7, Column 3Auristatin hydroxypropylamide (dolasynthen)Auristatin F-HydroxypropylamideSection 5.4.Trastuzumab-dolasynthenDolasynthen B7-H4 Directed ADC
